# The striatal-enriched protein Rhes is a critical modulator of cocaine-induced molecular and behavioral responses

**DOI:** 10.1038/s41598-019-51839-w

**Published:** 2019-10-25

**Authors:** Francesco Napolitano, Arianna De Rosa, Rosita Russo, Anna Di Maio, Martina Garofalo, Mauro Federici, Sara Migliarini, Ada Ledonne, Francesca Romana Rizzo, Luigi Avallone, Tommaso Nuzzo, Tommaso Biagini, Massimo Pasqualetti, Nicola Biagio Mercuri, Tommaso Mazza, Angela Chambery, Alessandro Usiello

**Affiliations:** 1 Department of Veterinary Medicine and Animal Productions, University of Naples Federico II, Naples, Italy; 2 CEINGE Biotecnologie Avanzate, Via G. Salvatore, 482, 80145 Naples, Italy; 3 IRCCS SDN, Via E. Gianturco 113, Naples, Italy; 4 Department of Environmental, Biological and Pharmaceutical Sciences and Technologies, University of Campania, “L. Vanvitelli”, Via A. Vivaldi 43, 81100 Caserta, Italy; 5 Fondazione Santa Lucia IRCCS, Via del Fosso di Fiorano 64, 00143 Rome, Italy; 6 Unit of Cell and Developmental Biology, Department of Biology, University of Pisa, Pisa, Italy; 7 University of Rome ‘Tor Vergata’, 00133 Rome, Italy; 8 Bioinformatics Unit, Fondazione IRCCS Casa Sollievo della Sofferenza, 71013 San Giovanni Rotondo, Foggia, Italy; 9 Center for Neuroscience and Cognitive Systems @UniTn, Istituto Italiano di Tecnologia, Rovereto, Italy

**Keywords:** Molecular neuroscience, Addiction

## Abstract

Previous evidence pointed out a role for the striatal-enriched protein Rhes in modulating dopaminergic transmission. Based on the knowledge that cocaine induces both addiction and motor stimulation, through its ability to enhance dopaminergic signaling in the corpus striatum, we have now explored the involvement of Rhes in the effects associated with this psychostimulant. Our behavioral data showed that a lack of Rhes in knockout animals caused profound alterations in motor stimulation following cocaine exposure, eliciting a significant leftward shift in the dose-response curve and triggering a dramatic hyperactivity. We also found that Rhes modulated either short- or long-term motor sensitization induced by cocaine, since lack of this protein prevents both of them in mutants. Consistent with this *in vivo* observation, we found that lack of Rhes in mice caused a greater increase in striatal cocaine-dependent D1R/cAMP/PKA signaling, along with considerable enhancement of Arc, zif268, and Homer1 mRNA expression. We also documented that lack of Rhes in mice produced cocaine-related striatal alterations in proteomic profiling, with a differential expression of proteins clustering in calcium homeostasis and cytoskeletal protein binding categories. Despite dramatic striatal alterations associated to cocaine exposure, our data did not reveal any significant changes in midbrain dopaminergic neurons as a lack of Rhes did not affect: (i) DAT activity; (ii) D2R-dependent regulation of GIRK; and (iii) D2R-dependent regulation of dopamine release. Collectively, our results strengthen the view that Rhes acts as a pivotal physiological “molecular brake” for striatal dopaminergic system overactivation induced by psychostimulants, thus making this protein of interest in regulating the molecular mechanism underpinning cocaine-dependent motor stimulatory effects.

## Introduction

The potent hard drug cocaine, deriving from the coca bush (*Erythroxylum coca*) leaf, functions by blocking the dopamine transporter (DAT), thereby triggering a dramatic increase in extracellular dopamine levels within corpus striatum, which is thought to be instrumental for its addictive and motor stimulatory properties^1,2^. In particular, it has been shown that the dorsal striatum (DStr) and the ventral striatum, also referred to as nucleus accumbens (NAc), play pivotal roles in the motor and hedonic effects evoked by cocaine^3,4^. Anatomically, the DStr and NAc are mainly composed of GABAergic medium spiny neurons (MSNs), that are segregated into the direct and indirect output pathways of the basal ganglia, determined by the expression of either dopamine D1 or D2 receptors (D1R or D2R), respectively^5,6^. Although extensive literature has clearly demonstrated the primary involvement of striatal D1R and D2R transmission in mediating the effects of cocaine, the nature and role of their specific pathways downstream of dopamine receptor activation still remain unclear^7–11^. Consistently, considerable amount of efforts has been devoted to identifying and characterizing genes encoding for proteins selectively expressed in the dopaminoceptive neurons of DStr and the NAc^12,13^. One gene of particular interest, termed “Ras homolog enriched in striatum” (Rhes), which encodes a small GTPase highly abundant in the corpus striatum, is expressed in virtually all dopamine D1R- and D2R-bearing MSNs, as well as in large aspiny cholinergic interneurons (ChIs) of rodent and human brains^13–18^. In addition to its striatal-enriched expression, Rhes mRNA is also found to a lesser extent in midbrain dopaminergic neurons of the substantia nigra *pars compacta* (SNc) and ventral tegmental area (VTA)^19^. In line with neuroanatomical data, which overall highlight the presence of the Rhes transcript in the vast majority of dopaminoceptive neurons, several investigations indicate that this protein modulates D1R and D2R signaling^20^. Accordingly, it has been reported that Rhes regulates D2R-related function in MSNs, either by directly affecting Go/i coupling, as measured by [^35^S]GTPγS binding assay^14^, or by modulating the activity of adenosine A2A receptor, known to exert antagonistic interaction upon D2R signaling^21,22^. In contrast, a lack of Rhes causes profound alterations in the excitability of striatal cholinergic interneurons, an effect explained by the property of Rhes to regulate the PI3K/Akt signaling pathway in these neurons downstream of D2R stimulation^16,20^. Furthermore, evidence obtained by *in vitro* and *ex vivo* studies indicate that Rhes modulates D1R/cAMP/PKA signaling directly upstream the activation of the heterotrimeric G-protein complex^23,24^.

In agreement with this evidence, recent studies reported that lack of Rhes in mice enhanced the motor stimulation associated to amphetamine^17^, phencyclidine^17^, MDMA^25^ administration, suggesting a primary role of this protein in modulating psychostimulants responses.

Here, to further extend the knowledge on the influence of Rhes in regulating drug of abuse effects, we investigated the involvement of this striatal protein in controlling motor stimulant and hedonic properties associated with cocaine exposure.

## Results

### Rhes controls motor stimulant effect induced by cocaine

**Figure 1:**
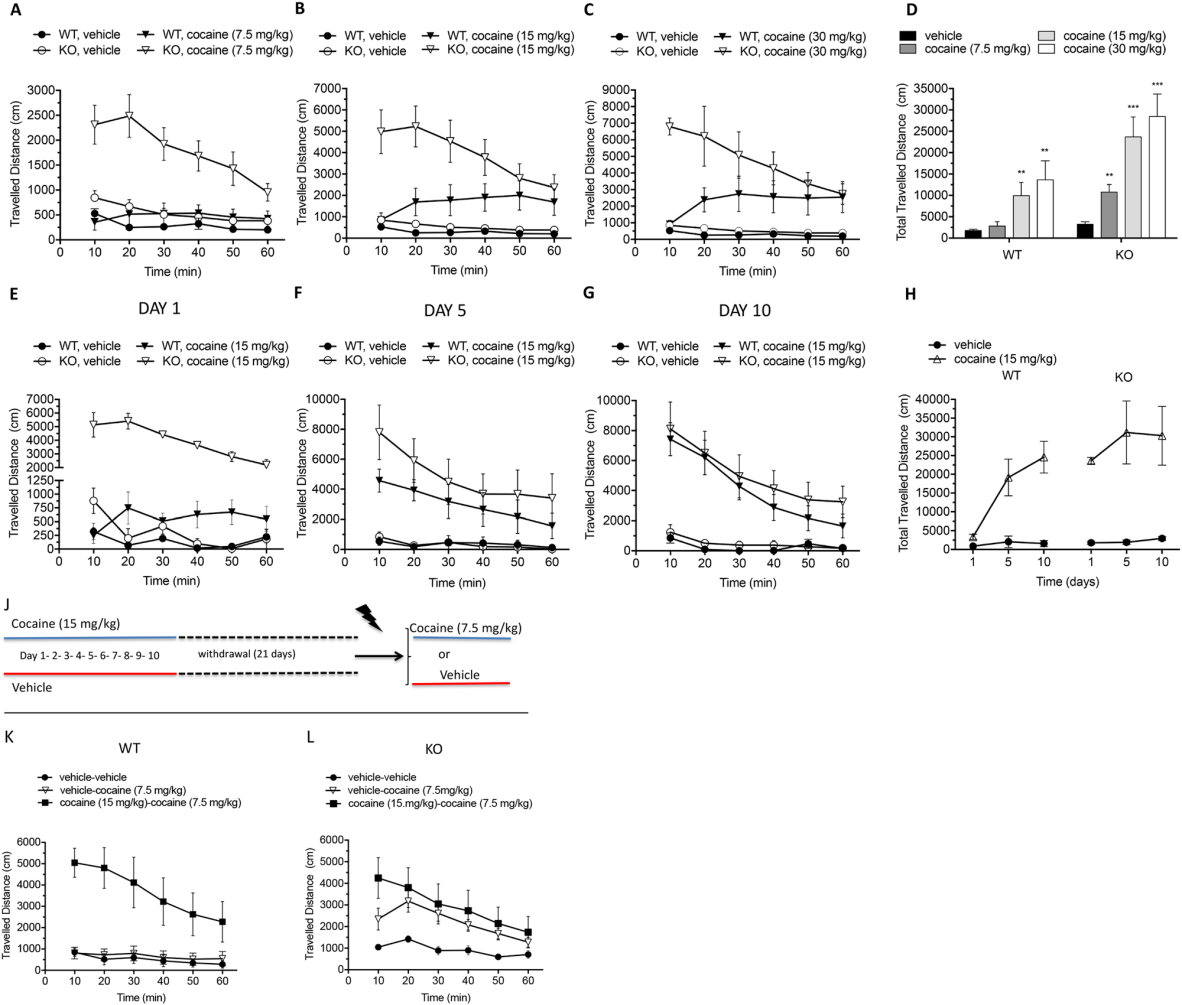
Effect of cocaine treatment on locomotor activity. (**A**–**D**) Horizontal motor activity induced by acute intraperitoneal administration of 7.5 (n = 11/genotype) (**A**), 15 (n = 11 WT, 10 KO) (**B**), and 30 mg/kg cocaine (n = 5/genotype) (**C**) or vehicle (n = 19 WT, 18 KO). (**E**–**H**) Effect of repeated cocaine treatment on locomotor activity. Horizontal motor activity at day 1 (**E**), 5 (**F**), and 10 (**G**) following i.p. administration of 15 mg/kg cocaine (n = 6/genotype) or vehicle (n = 3/genotype). (**J**–**L**) Evaluation of the horizontal motor activity upon intermittent cocaine treatment. (**J**) Timeline and experimental design. (**K**,**L**) Locomotor activity data from WT (**K**) and KO (**L**) animals receiving repeated 15 mg/kg cocaine or vehicle injections (day 1–10). After 21-day withdrawal, vehicle-treated group was challenged with 7.5 mg/kg cocaine (vehicle-cocaine group: n = 8/genotype) or vehicle (vehicle-vehicle group: n = 9/per genotype), while cocaine-treated mice were given 7.5 mg/kg cocaine (cocaine-cocaine group: n = 10/per genotype). Locomotion is expressed as distance traveled (cm), measured every 10 min over a 60-min test session, and as total traveled distance (D and H). **p < 0.01, ***p < 0.0001 vs vehicle-treated group within genotype, Uncorrected Fisher’s LSD test. All values are expressed as mean ± SEM. Genotypes and treatments are as indicated.

First, we tested the influence of Rhes in modulating locomotor response induced by the acute administration of cocaine at different concentrations (7.5, 15, or 30 mg/kg). Two-way RM ANOVA showed that a cocaine-dependent hyperlocomotion occurred in KO animals at the lowest dose tested (7.5 mg/kg), while no changes were observed in WT-treated mice, when compared to their vehicle-treated controls (WT: F_(1,28)_ = 1.439, *p* = 0.2403; KO: F_(1,27)_ = 24.48, *p* < 0.0001; Fig. 1A). In contrast, both 15 and 30 mg/kg cocaine caused a significant motor stimulation in all animals (15 mg/kg, WT: F_(1,28)_ = 11.85, *p* = 0.0018; KO: F_(1,26)_ = 34.15, *p* < 0.0001; 30 mg/kg, WT: F_(1,22)_ = 28.73, *p* < 0.0001; KO: F_(1,21)_ = 79.66, *p* < 0.0001; Fig. 1B,C), although with a greater response in mutants (three-way RM ANOVA; genotype x treatment interaction, 15 mg/kg: F_(1,270)_ = 8.718, *p* = 0.0047; 30 mg/kg: F_(1,215)_ = 13.947, *p* = 0.0005). Notably, 15 and 30 mg/kg cocaine caused a different time course of motor stimulation between genotypes (three-way RM ANOVA; genotype x treatment x time course interaction, 15 mg/kg: F_(5,270)_ = 9.336, *p* < 0.0001; 30 mg/kg: F_(5,215)_ = 17.964, *p* < 0.0001). Overall, the greater sensitivity and magnitude of cocaine-dependent hyperlocomotion in KO mice at all doses tested was also confirmed by analyzing the total distance traveled (Fig. 1D). Then, we investigated the influence of Rhes in regulating the locomotor effect associated with repeated cocaine exposure. Accordingly, the animals received 15 mg/kg cocaine once a day for 10 consecutive days and the distance traveled was evaluated on days 1, 5, and 10. Similarly to what previously observed (Fig. 1B), the single cocaine injection (day 1) induced hyperlocomotion in both genotypes, although the magnitude was greater in KO mice (Three-way RM ANOVA genotype x treatment interaction: F_(1,70)_ = 138.380, *p* < 0.0001; Fig. 1E). Interestingly, as cocaine administration progressed, we failed to find any main genotype effect in either magnitude of motor stimulation, or pharmacokinetic profile of cocaine (genotype x treatment interaction, day 5: F_(1,70)_ = 0.506, *p* = 0.4887; day 10: F_(1,70)_ = 0.034, *p* = 0.8651; genotype x treatment x time course interaction, day 5: F_(5,70)_ = 0.425, *p* = 0.8295; day 10: F_(5,70)_ = 0.225, *p* = 0.9506; Fig. 1F,G). Overall, our behavioral data highlight a primary role of Rhes in controlling motor stimulation under both acute and chronic cocaine administration. Indeed, differently to WT mice statistical analysis indicated a no main effect of treatment x time interaction in KO, (two-way RM ANOVA; WT: F_(2,14)_ = 6.733, *p* = 0.0089; KO: F_(2,14)_ = 0.3054, *p* = 0.7416; Fig. 1H). Finally, we investigated the role of Rhes in modulating cocaine-dependent long-term motor sensitization^26^, analyzing the changes in psychostimulant-induced motor stimulation in response to 10 consecutive days of pretreatment either with drug (15 mg/kg) or with vehicle, followed by administration of a single low dose of cocaine (7.5 mg/kg) or vehicle after a 21-day withdrawal period, as described^27^ (Fig. 1J). As expected with this protocol, behavioral data showed the presence of a significant cocaine sensitization in drug-pretreated WT animals, as compared to vehicle-pretreated controls, that received 7.5 mg/kg cocaine (two-way RM ANOVA; cocaine-cocaine *vs* vehicle-cocaine groups, treatment x time course interaction: F_(5,80)_ = 2.549, *p* = 0.0342; Fig. 1K). Conversely, in KO mice we found that cocaine pretreatment failed to further enhance locomotor stimulation induced by a single administration of 7.5 mg/kg cocaine subsequent to 3-weeks of withdrawal period (F_(5,80)_ = 1.773, *p* = 0.1279; Fig. 1L). In contrast to acute injection, the time course of cocaine-induced motor stimulation in the WT-sensitized mice could not be distinguishable from that reported for acute treatment with cocaine in KO mice (Fig. 1B,C). Thus, our *in vivo* data suggested that Rhes has a relevant physiological role in regulating both the expression of cocaine-induced motor stimulation and time-course related to the motor effects associated to cocaine exposure.

### Lack of Rhes does not affect cocaine-induced conditioned place preference

**Figure 2:**
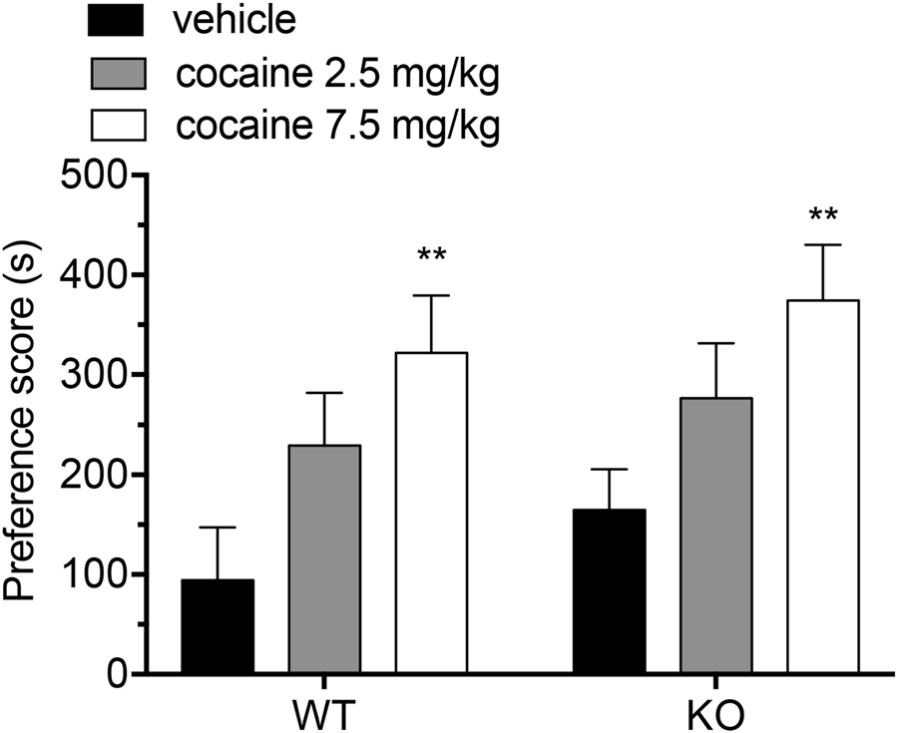
Cocaine-induced CPP in Rhes KO mice. Evaluation of the preference score (time spent in the drug-paired compartment – time spent in the pre-conditioning test) by treating the mice with cocaine at the dose of 2.5 mg/kg (n = 10/genotype), 7.5 mg/kg (n = 10/genotype) or vehicle (n = 11/genotype). All values are expressed as mean ± SEM. Genotypes and treatments are as indicated. **p < 0.01, ***p < 0.0001, compared to vehicle-treated group within genotype.

Here, we evaluated whether Rhes could modulate cocaine-induced rewarding properties, by performing conditioning place-preference (CPP) paradigm^11^ in KO mice and WT controls, treated with 2.5, 7.5 mg/kg cocaine or vehicle. Data, expressed as difference (in seconds) between the time spent in the cocaine-paired compartment and the time spent in the pre-conditioning test (preference score), showed that in both genotypes cocaine induced a comparable dose-dependent CPP. Indeed, two-way ANOVA analysis indicated a significant cocaine effect (F_(2,56)_ = 8.918, *p* = 0.0004), but not a main genotype effect (F_(1,56)_ = 1.774, *p* = 0.1883), nor genotype x treatment interaction (F_(2,56)_ = 0.027, *p* = 0.9733; Fig. 2). Thus, present findings indicate that lack of Rhes does not alter in mice the hedonic properties of cocaine as tested by CPP paradigm.

### Rhes in the dopaminergic neurons does not affect cocaine-induced dopamine release and D2R-mediated responses

**Figure 3:**
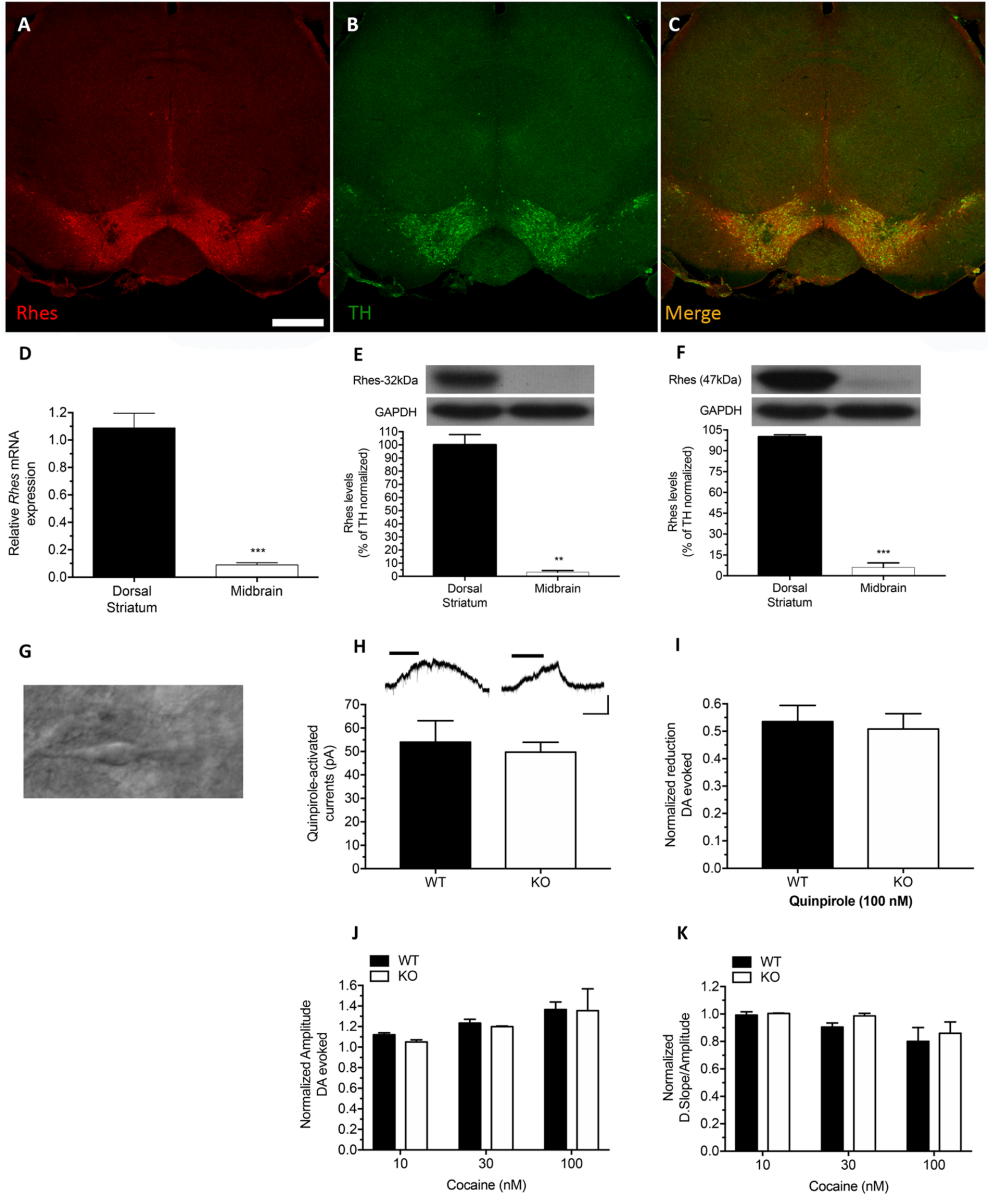
Lack of Rhes does not affect D2R-mediated responses in the dopaminergic neurons and cocaine-induced dopamine release in the striatum. (**A**–**C**) Representative confocal images of midbrain coronal section processed for combined *in situ* hybridization and immunohistochemical labeling showing coexpression of Rhes mRNA and TH protein in midbrain dopaminergic neurons. Scale bar: 500 μm. (**D**–**F**) Quantitative reverse transcription-PCR to evaluate Rhes mRNA expression in DStr (n = 6) and midbrain (n = 6) from WT mouse samples. ***p < 0.0001, compared to DStr group, Unpaired t test. (**E**,**F**) Analysis of 32-kDa (**E**) and 47-kDa (**F**) Rhes protein abundance in DStr and midbrain (n = 3) from wild-type mouse lysates. Unpaired t test, *p* < 0.001 (**E**), *p* < 0.0001 (**F**). ***p < 0.0001, compared to DStr grou*p*. (**G**) Infrared videomicroscopy image of a midbrain dopamine neuron selected for patch-clamp recordings. (**H**) Representative traces (upper panel) of quinpirole (1 µM, 3 min)-induced currents in midbrain dopamine neurons from brain slices of Rhes KO and WT littermates (n = 7) and histogram of mean amplitudes of quinpirole-activated currents. (**I**) Normalized reduction of the stimulus-evoked DA release by bath perfusion of quinpirole 100 nM (10 min) in WT and KO (n = 4) male mice. (**J**,**K**) CPA recordings showing the effect of cocaine in striatum on evoked dopamine in WT and KO (n = 3) male mice. (**J**) Effect of cocaine at the different concentrations [nM] on dopamine efflux, represented as normalized values of increased amplitude. (**K**) Effect of cocaine on the uptake rate, represented as normalized values of decay slope/amplitude. All values are expressed as mean ± SEM. Genotypes and treatments are as indicated.

Here, we sought to investigate whether the abnormal cocaine-induced motor stimulation in the Rhes KO might be due to altered presynaptic functions underlying dopamine release in the striatum of mutants. Initially, we confirmed that the Rhes transcript was expressed in midbrain neurons by applying a combined ISH and IHC approach (Fig. 3A–C). Then, qPCR experiments revealed that Rhes mRNA expression in the dorsal striatum was dramatically higher (>12 fold) than in the midbrain region (Unpaired t test: *p* < 0.0001; Fig. 3D). Consistent with these data, western blotting experiments indicated that both 32- and 47-kDa Rhes protein isoforms were abundantly expressed in the dorsal striatum, while their presence in the midbrain was barely detectable, with 30- and 20-fold lower amounts than in the DStr, respectively (Unpaired t test, 32 kDa isoform: *p* = 0.0003; 47 kDa isoform: *p* < 0.0001; Fig. 3E,F).

Based on the influence of presynaptic regulation of dopamine release in regulating cocaine response^28,29^, we investigated whether Rhes could regulate D2R functioning in mesencephalic dopaminergic neurons, either at somatodendritic or at nerve terminal levels. Accordingly, we performed electrophysiological patch-clamp recordings of nigral dopamine neurons (Fig. 3G) from KO mice and WT littermates, by analyzing the effects of the selective D2R agonist quinpirole in modulating Gi/o–gated K^+^ (GIRK) channels. In mesencephalic dopaminergic neurons the activation of D2 receptors induces GIRK channel opening, thus producing outward currents^30^. We found that membrane currents induced by applying quinpirole (1 µM, 3 min) were similar among the experimental groups, thus implying that a lack of Rhes expression did not significantly affect D2R-dependent GIRK channel opening in mesencephalic dopaminergic neurons (Unpaired t test: *p* > 0.9999; Fig. 3H). In addition, we explored the role of Rhes in regulating D2R-dependent modulation of dopamine release at nerve terminals. Correspondingly, we tested the neurochemical effect of quinpirole in regulating striatal dopamine release as measured by voltammetry. We found that quinpirole (100 nM) was able to reduce the amperometric signal that is due to the same extent of DA release in KO mice and WT littermates (Unpaired t test: *p* = 0.749; Fig. 3I). Finally, we sought to explore whether a lack of Rhes in mice could influence the cocaine effect upon DAT activity. The amperometric measurements showed that, at doses between 10 and 100 nM, cocaine produced a comparable enhancement of the DAergic signal in the striatum of both genotypes. Unpaired t test revealed no statistical differences between genotypes for either the amplitude (10 nM: *p* = 0.0694; 30 nM: *p* = 0.4171; 100 nM: *p* = 0.9621) or time of dopamine reuptake (D.Slope/Amp) (10 nM: *p* = 0.6470; 30 nM: *p* = 0.0760; 100 nM: *p* = 0.6705; Fig. 3J,K). Thus, present findings indicate that Rhes does not alter either DAT activity or D2R-dependent regulation of dopamine release in the striatum.

### Rhes affects cocaine-related D1R/cAMP/PKA signaling and immediate early gene expression in the striatum

**Figure 4:**
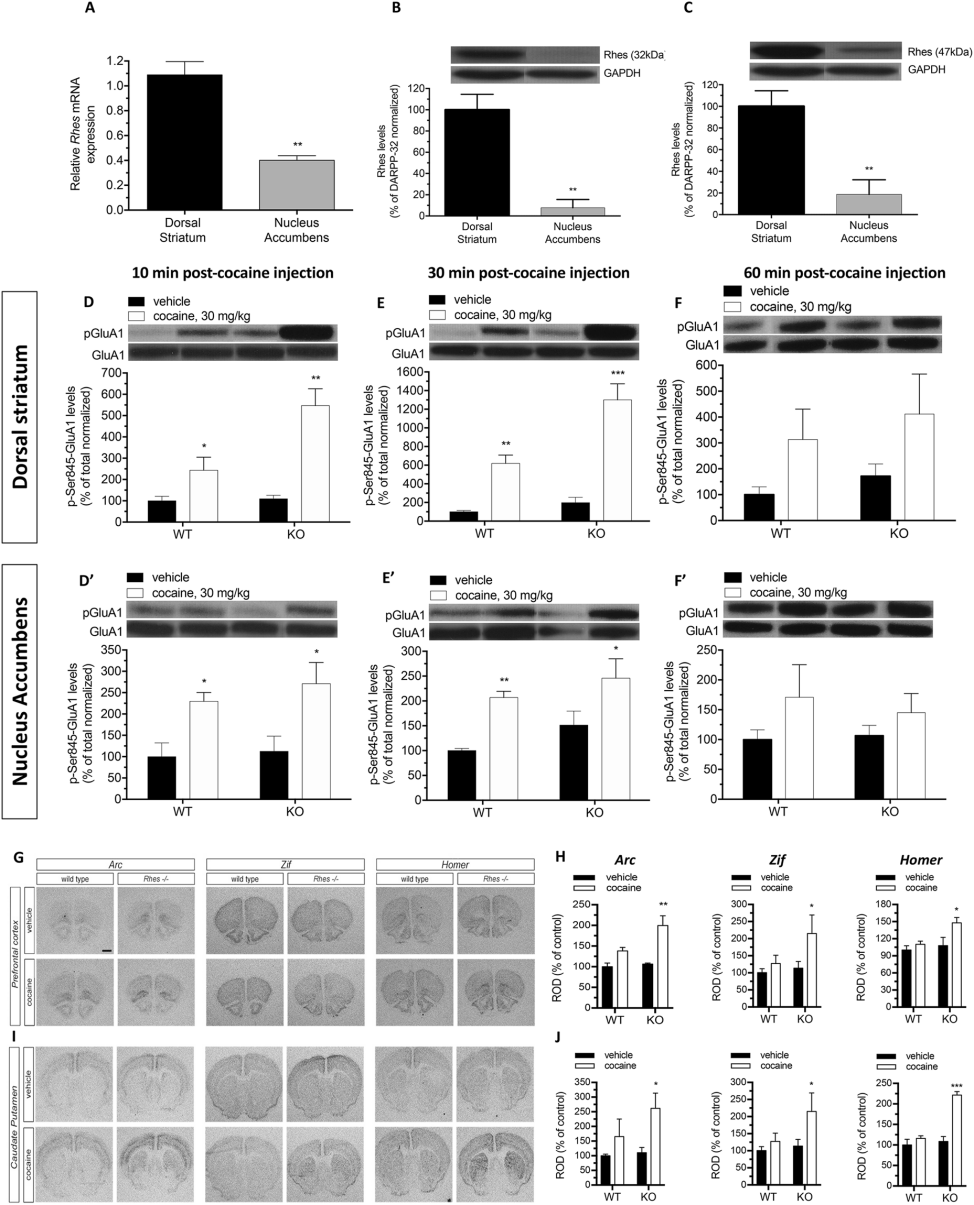
Role of Rhes in the modulation of cocaine induced-cAMP/PKA pathway activation in DStr and NAc and in the induction of the immediate-early gene expression. (**A**) Quantitative reverse transcription-PCR to evaluate Rhes mRNA expression in DStr (n = 6) and NAc (n = 6) from wild-type mouse samples. (**B**,**C**) Analysis of 32 kDa (**B**) and 47 kDa (**C**) Rhes protein abundance in DStr (n = 5) and NAc (n = 4) from wild-type mouse lysates. Unpaired t test. **p < 0.01, compared to DStr group. (**D**–**F**) Evaluation of pGluA1protein levels, following 30 mg/kg cocaine treatment in WT and KO mice at different time points post-cocaine injection in DStr (10 min: vehicle, n = 6 WT/3 KO; cocaine, n = 6 WT/4 KO; 30 min: vehicle, n = 5/genotype; cocaine, n = 5/genotype; 60 min: vehicle, n = 4/genotype; cocaine, n = 5/genotype) (**D**–**F**) and NAc (10 min: vehicle, n = 6 WT/3 KO; cocaine, n = 6/genotype; 30 min: vehicle, n = 5/genotype; cocaine, n = 5/genotype; 60 min: vehicle, n = 4/genotype; cocaine, n = 5/genotype) (**D'**–**F'**). *p < 0.05, **p < 0.01, ***p < 0.0001, compared to vehicle-treated group within genotype, Uncorrected Fisher’s LSD test. Top panels show representative blots. All representative blots shown in the figures arise from cut-out and pasted bands for reassembling the image. Of note, the representative bands come from the same films for each graph. G,I Representative images of radioactive *in situ* hybridization (ISH) on mouse prefrontal cortex (**G**) and caudate putamen (**I**) using specific antisense riboprobes for the immediate-early genes *Arc*, *Zif 268*, and *Homer 1a*. Densitometric analysis of autoradiograms reported in the histogram show the quantification of *Arc*, *Zif 268*, and *Homer 1a* expression levels in the prefrontal cortex (**H**) and caudate putamen (**J**) of adult wild-type and KO mice treated with cocaine or vehicle. Scale bar 500 μm. *p < 0.05, **p < 0.01, compared to vehicle-treated group within genoty*p*e, Uncorrected Fisher’s LSD test. Values are expressed as mean ± SEM of relative optical density (ROD). Genotypes and treatments are as indicated.

Based on the evidence that motor-stimulant properties of cocaine primarily stem from molecular events occurring in the dorsal and ventral striatum, we initially intended to evaluate the yet unknown selective Rhes mRNA and protein expression within these brain regions. In line with earlier northern blot and ISH investigations^13,24^, we confirmed by qPCR analysis here that the Rhes transcript was expressed in the whole striatum, although its regional abundance was about 3-fold greater in the DStr than in the NAc (Unpaired t test: *p* = 0.0001; Fig. 4A). We also reported by western blotting that protein levels of both the 32- and 47-kDa Rhes isoforms were, respectively, 12- and 5-fold higher in the DStr than in the NAc (Unpaired t test; 32 kDa isoform: *p* = 0.0013; 47 kDa isoform: *p* = 0.005, Fig. 4B,C). Then, to discover whether changes in D1R/cAMP/PKA signaling, which is known to strictly control the motor-stimulant effect of cocaine^31–33^, correlate with the greater hyperlocomotion elicited by cocaine exposure in mutants, we investigated the involvement of Rhes in modulating this signaling pathway under cocaine exposure in both DStr and NAc. Western blotting results showed that cocaine caused an overall increase in the PKA-dependent GluA1 phosphorylation state at the Ser845 residue (pGluA1) in DStr 10 and 30 min after injection in males of both genotypes, although with a more pronounced effect in KO mice (Two-way ANOVA, 10 min: F_(1,15)_ = 27.3, *p* = 0.0001; 30 min: F_(1,16)_ = 63.77, *p* < 0.0001; genotype x treatment interaction; 10 min: F_(1,15)_ = 6.965, *p* = 0.0186; 30 min: F_(1,16)_ = 8.261, *p* = 0.0110; Fig. 4D,E). On the other hand, we found similar GluA1 hyperphosphorylation between genotypes 10 and 30 min after injection in the NAc of cocaine-treated mice (Two-way ANOVA, 10 min: F_(1,17)_ = 13.57, *p* = 0.0018; 30 min: F_(1,16)_ = 16.22, *p* = 0.0010; genotype x treatment interaction, 10 min: F_(1,17)_ = 0.1347, *p* = 0.7181; 30 min: F_(1,16)_ = 0.05982, *p* = 0.8099; Fig. 4D',E'). No main differences in cAMP/PKA activity were found between genotypes and brain regions analyzed 60 min after cocaine exposure (Two-way ANOVA, DStr: F_(1,14)_ = 4.044, *p* = 0.0640; NAc: F_(1,14)_ = 2.148, *p* = 0.1649; Fig. 4F,F'). Therefore, according to Rhes protein expression data, these findings reveal that Rhes mediates cocaine-induced pGluA1 phosphorylation in the DStr to a much greater extent than in the NAc.

It is well established that cocaine can modify chromatin structure via its ability to increase dopamine D1R-dependent signaling^34,35^ and stimulate striatal and cortical neuronal activity which, in turn, is mirrored by a substantial expression of the immediate early genes (IEGs)^33,36,37^. Here, employing the same set of KO and WT male mice that received either 15 mg/kg of cocaine or vehicle (Fig. 1B), we performed quantitative ISH using ^35^S-radiolabeled antisense probes for Arc, Zif 268, and Homer 1a after 90 min of drug treatment. Notably, densitometric quantification of autoradiograms showed an overall greater cocaine-dependent increase in Arc, Zif 268, and Homer 1a mRNA levels in both prefrontal cortex and DStr of KO mice as compared to WT-treated animals (Uncorrected Fisher’s LSD; prefrontal cortex, Arc; WT: *p* = 0.3052, KO: *p* = 0.0005; Zif, WT: *p* = 0.5274, KO: *p* = 0.0422; Homer, WT: *p* = 0.4776, KO: *p* = 0.0139, Fig. 4G,H; DStr, Arc; WT: *p* = 0.2642, KO *p* = 0,0131; Zif, WT: *p* = 0.5502, KO: *p* = 0.0426; Homer, WT: *p* = 0.3052, KO: *p* < 0.0001; Fig. 4I,J).

### Rhes modulates protein expression in dorsal striatum upon cocaine administration

**Figure 5:**
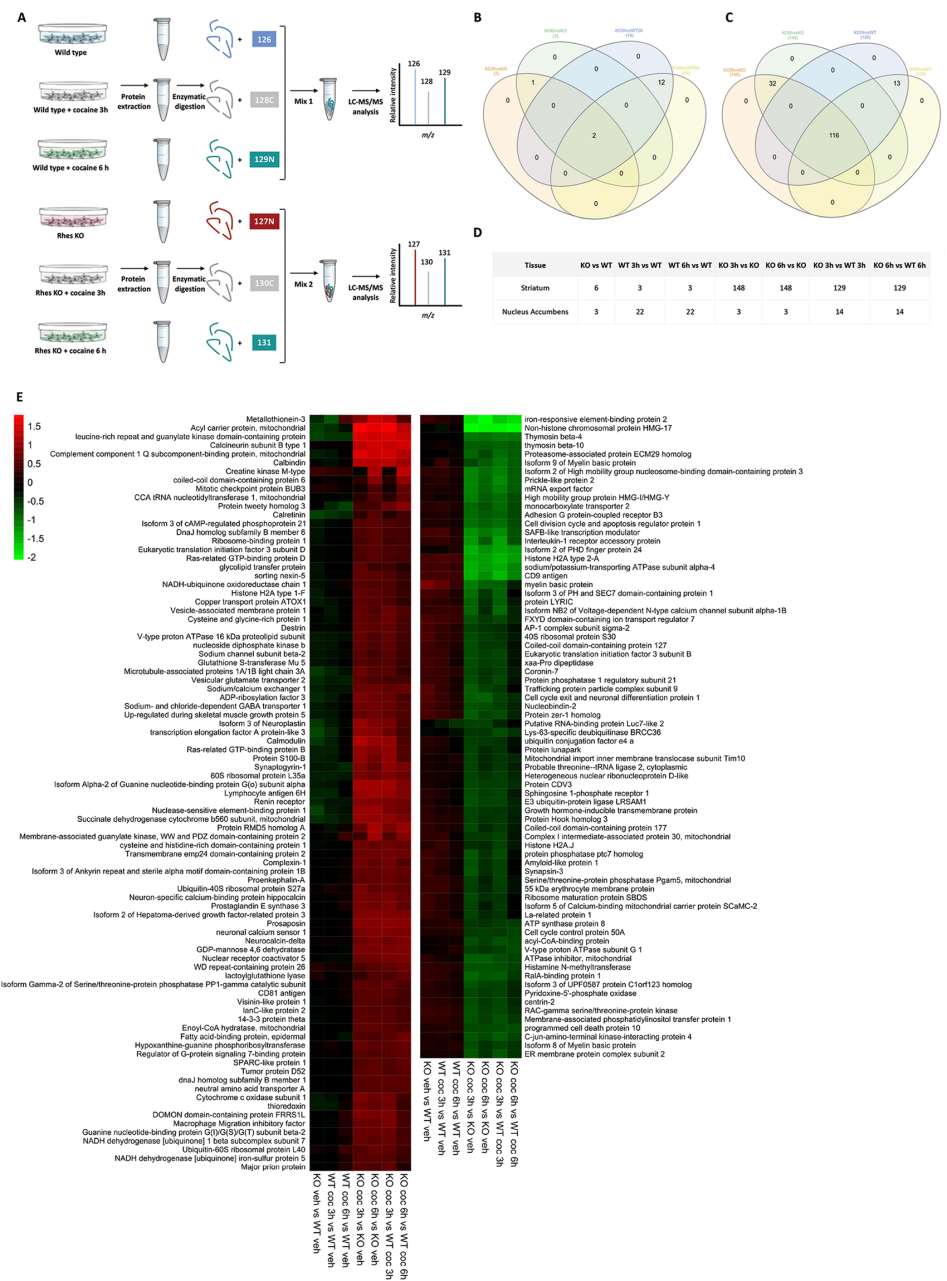
Quantitative proteomic analyses by high resolution nano LC-MS/MS. (**A**) Schematic overview of the experimental workflow used for the quantitative proteomic analyses by high resolution nano LC-MS/MS. A high number of peptide groups (i.e., 31012 and 29547 for DStr and NAc tissues, respectively) was used for protein identification and, out of these, more than 80% were used as unique peptides for protein quantification, attesting the high efficiency of peptide labeling (data not shown). (**B**,**C**) Venn diagrams showing the overlapping of proteins differentially expressed in NAc (**B**) and DStr (**C**) tissues under different conditions. (**D**) Compared conditions together with numbers of differentially expressed proteins. (**E**) Heatmap of differentially expressed proteins in DStr tissue samples of WT and KO mice. Down-regulated and up-regulated proteins are colored in green and red respectively. Colour gradients represent the strength of differential expression.

In order to further investigating the role of Rhes in modulating cocaine-dependent responses in MSNs, protein expression analysis in KO and WT mice following cocaine treatment was measured by a quantitative proteomic approach^38^. The schematic representation of the experimental design applied on both DStr and NAc tissues is depicted in Fig. 5A. We identified and quantified 2474 and 2681 nonredundant proteins, with more than one peptide in at least two out of three injections in DStr and NAc samples, respectively (data not shown). About 78% of proteins were commonly identified in both tissues, while only about 7% and 15% of proteins were found to be uniquely identified in NAc and DStr tissues, respectively (Fig. 5B,C). Moreover, small fractions were found differentially expressed in both DStr (6.5%) and NAc (1%) samples in at least one out of the seven comparisons (Fig. 5D). A small number of proteins were differentially expressed in NAc samples in all analyzed ratios (Fig. S1). In contrast, most proteins differentially expressed in the DStr were only found in KO samples treated with cocaine at both 3 h and 6 h (Fig. 5E).

The functional enrichment analysis of these proteins performed revealed that transport of molecules and ions, cytoskeletal modification, movement disorders, dyskinesia, and cell death were among the top enriched diseases and functions categories (Fig. S2). It should be noted that specific subsets of differentially expressed proteins in Rhes KO mice treated with cocaine at 3 h and 6 h with respect to wild-type samples were mapped on pathways converging on ERK. Similar network maps were observed when comparisons were performed with respect to the untreated KO sample (Fig. S2B,C).

## Discussion

Here, we explored the presynaptic and postsynaptic role of Rhes in modulating behavioral, signaling, gene and protein expression consequences associated with cocaine administration in mice. Remarkably, our results documented that Rhes acts as a physiological negative modulator of striatal dopaminergic system overactivation induced by cocaine. In line with this assumption, we found that a lack of Rhes in KO mice triggered profound behavioral alterations in the time-course of cocaine-dependent motor stimulation, with a significant leftward shift in the dose-response curve, and an abnormally higher hyperactivity than WT-treated mice. Moreover, Rhes also regulates the expression of short-term locomotor sensitization induced by repeated administrations of cocaine. We also documented that a lack of Rhes impacts on the long-lasting cocaine-dependent locomotor sensitization since, differently to what observed in WT-treated mice, the higher cocaine-dependent motor activity seen in mutants didn’t allow us to detect any further enhancement of locomotion, either under a chronic or intermittent schedule of drug administration. One of the reasonable explanations of this phenomenon in KO mice might be due to a “ceiling effect” in cocaine-dependent motor stimulation found in mutants since the first drug exposure.

Consistent with accumulating studies showing a strict link between motor stimulatory properties of psychostimulants and striatal cAMP/PKA signaling pathway activation downstream dopamine D1R-dependent activity^33,39,40^, we found that cocaine exposure caused a greater increase in the phosphorylation state of GluA1 at the Ser845 residue (one of the main striatal PKA substrates) in the DStr of KO mice, compared to WT-treated controls. Thus, in agreement with previous reports^14,15,23,41^, the present observations suggest that Rhes physiologically plays an inhibitory role in counteracting the enhancement of striatal cAMP/PKA signaling downstream dopamine D1R activation induced by increased DA levels associated to cocaine exposure. Interestingly, pGluA1 levels in the NAc of cocaine-treated mice were similarly increased in both genotypes. To explain this apparent discrepancy, we argue that the low levels of Rhes protein in NAc would prevent us from appreciating any detectable contribution of this GTPase in modulating cocaine-dependent effects upon cAMP/PKA signaling, as measured by western blot analysis. On the other hand, based on the knowledge that cocaine-dependent increase in striatal D1R/cAMP/PKA signaling triggers transient activation of IEGs in the corticostriatal circuitry^42,43^, we explained the greater increase of Arc, zif268, and Homer1 transcript levels, in both prefrontal cortex and DStr of KO mice, when compared to WT-treated mice.

Here, we also explored the influence of Rhes in regulating the striatal protein expression after cocaine injection. We reported that a lack of Rhes in mice elicit profound striatal alterations of the proteomic signature following cocaine exposure and this effect was much more pronounced in the DStr than in NAc. Coherently with remarkable influence of cocaine exposure in triggering widespread neuronal activation and synaptic modifications^44,45^, most of the differentially expressed molecules in the DStr of KO mice were clustered in the calcium influx, calcium ion binding, and cytoskeletal protein binding categories. Hence, given the primary role of calcium-stimulated second messengers in the expression of striatal synaptic modifications associated with cocaine exposure, we hypothesize that abnormal modifications in calcium signaling found in treated KO animals may represent an essential biochemical substrate through which the transient neurochemical changes in dopamine transmission are translated into persistent molecular adaptations in the DStr of mutants. Furthermore, these data are in line with previous reports documenting that Rhes, by influencing Gαi-coupled GPCR signaling, negatively modulates the activity of the voltage-dependent Cav2.2 (N-type) calcium channels^16,46^. Importantly, a previous IP/MS/MS study showed that Rhes rapidly forms the protein complex “Rhesactome” in the striatum immediately after administering the dopamine releaser amphetamine, thus indicating that this protein complex may be required for motor regulatory role of Rhes in psychostimulant-related effects^47^. Therefore, Rhes, in addition to influencing striatal protein-protein interaction as reported by Subramaniam and colleagues^47^, can directly alter the dopamine-dependent protein expression in the striatum, as reported here by a quantitative proteomic approach. Anyway, whether Rhes forms a similar “Rhesactome” complex upon cocaine administration remains to be determined.

Of interest, we reported that a lack of Rhes failed to significantly affect somatodendritic D2R-dependent regulation of GIRK and dopamine release at nerve terminals, as demonstrated by responses to the D2R-like agonist quinpirole via amperometry in the striatum, and patch-clamp recordings of dopaminergic neurons within the substantia nigra. Furthermore, amperometric investigations also ruled out an influence of Rhes in modulating DAT activity, since the influence of cocaine on dopamine release could not be distinguished in KO and control mice. Thus, we argue that Rhes has a specific influence in orchestrating cocaine-dependent molecular and behavioral motor effects by modulating striatal dopaminergic transmission at postsynaptic level.

Beyond its involvement in GPCR-mediated signaling, Rhes also binds to and activates the mTORC1 pathway^20^, which has been reported to be implicated in L-DOPA-induced dyskinesia (LID)^48–50^. Accordingly, a lack of Rhes in a mouse model of Parkinson’s disease significantly attenuates the expression of LID by reducing the striatal mTORC1 signaling^51^. Considering that both LID and psychostimulant-induced motor stimulation stem on the critical activation of striatal D1/cAMP/PKA-signaling^33,52^, here we ruled out that Rhes modulates cocaine-dependent motor responses through a mTORC1-dependent mechanism, since pretreatment in mutants with the mTORC1 inhibitor, rapamycin, did not affect the exaggerated acute cocaine-dependent hyper-locomotion (Fig. S3).

In conclusion, considering previous and present data indicating a pivotal role of Rhes in regulating the motor stimulant responses induced by amphetamine, phencyclidine, MDMA and cocaine exposure, our findings substantiate the general idea that Rhes acts in the striatum as a potent physiological molecular “brake” under over-activation of dopaminergic transmission, thus rendering this striatal-enriched protein a major striatal molecular candidate involved in the molecular events underpinning the motor stimulatory properties associated to psychostimulants abuse.

## Material and Methods

### Animals and drugs

For all details about animals see Supplementary Material. Experiments were carried out conformed to protocols approved by the veterinary department of the Italian Ministry of Health (Authorization number: 387/2017-PR of the Decree Law No. 26/2014-Implementation of the Directive 2010/63/EU about the protection of animals used for scientific purposes). Cocaine hydrochloride (Sigma–Aldrich, St. Quentin Fallavier, France) was dissolved in a 0.9% NaCl (w/v) aqueous solution (vehicle) and was administered by intraperitoneal injection (i.p.). Quinpirole hydrochloride (Sigma–Aldrich, St. Quentin Fallavier, France) was bath-applied (100 nM) on the striatal slices.

### Motor responses induced by cocaine treatment

Cocaine-induced locomotor hyperactivity was induced according to a previous protocol^11,53,54^ (See Supplementary Information).

#### RNA extraction

Total RNA was extracted by using the RNeasy mini kit (QIAGEN, Hilden, Germany) according to the manufacturer’s instructions. The integrity of the RNA was assessed by denaturing agarose gel electrophoresis (presence of sharp 28S, 18S, and 5S bands) and spectrophotometry (NanoDrop 2000, Thermo Scientific, Massachusetts, USA) (See Supplementary Information for RT-qPCR analysis).

### Cocaine conditioned place preference (CPP)

WT and KO male mice, treated with cocaine (2.5 mg/kg, 7.5 mg/kg) or vehicle, were tested for CPP protocol as previously reported^55^. For further detail see Supplementary Information.

### Western blotting

WT and KO male mice treated with cocaine (30 mg/kg) or vehicle were killed at 10, 30, and 60 min after injection and their heads immediately frozen in liquid nitrogen. Micropunches were sonicated in 1% SDS and boiled for 10 min (See Supplementary Information).

### Midbrain slice preparation

Acute midbrain slices used in the electrophysiological experiments were obtained by following standard procedures^56^ (See Supplementary Information).

### Electrophysiology

Whole-cell patch-clamp recordings of dopaminergic neurons from the SNpc and VTA of males were performed at 33.0 ± 0.5 C° in a recording chamber placed on the stage of an upright microscope (Axioscope FS, Zeiss, Gottingen, Germany) equipped for infrared video microscopy (Hamamatsu, Tokyo) (See supplementary Information).

### *In situ* hybridization

Male mice that received acute cocaine treatment at 15 mg/kg and analyzed for motor activity (Fig. 1B) were sacrificed at 90 min and used for ISH studies (See Supplementary Information).

### Sample preparation for quantitative proteomic analyses

Samples for proteomic analysis were prepared on DStr and NAc tissue sections collected from male WT and KO mice (pool of 4 animals), untreated or treated with cocaine at 3 h and 6 h (See Supplementary Information).

### Statistical analysis

Data were statistically evaluated by using a mix model of ANOVA. All Statistical analysis was performed with GraphPad (version 7.0; La Jolla, CA) and StatView softwares.

## Supplementary information

Napolitano et al-Supplementary information
