# Supplementary material for: The striatal-enriched protein Rhes is a critical modulator of cocaine-induced molecular and behavioral responses
Source: Sci Rep. 2019 Oct 25;9:15294. doi: 10.1038/s41598-019-51839-w (PMC6814836; doi:10.1038/s41598-019-51839-w)
Supplement: Supplementary file 1 — Napolitano et al-Supplementary information [file 41598_2019_51839_MOESM1_ESM.doc]

SUPPLEMENTARY ONLINE MATERIAL

**The striatal-enriched protein Rhes is a critical modulator of cocaine-induced molecular and behavioral responses**

Francesco Napolitano^1,2,#,*^, Arianna De Rosa^3,#^, Rosita Russo^4#^, Anna Di Maio^3,#^, Martina Garofalo^2,4^, Mauro Federici^5^, Sara Migliarini^6^, Ada Ledonne^5^, Francesca Romana Rizzo^7^, Luigi Avallone^1^,Tommaso Nuzzo^2,4^, Tommaso Biagini^8^, Massimo Pasqualetti^6,9^, Nicola Biagio Mercuri^7^, Tommaso Mazza^8^, Angela Chambery^4^, Alessandro Usiello^*2,3,4^

^1^Department of Veterinary Medicine and Animal Productions, University of Naples Federico II, Naples, Italy;

^2^CEINGE Biotecnologie Avanzate, Via G. Salvatore, 482, 80145, Naples, Italy;

^3^Istituto di Ricovero e Cura a Carattere Scientifico (IRCCS) Foundation SDN, Via Gianturco, Naples, Italy;

^4^Department of Environmental, Biological and Pharmaceutical Sciences and Technologies, University of Campania, “L. Vanvitelli”, Via A. Vivaldi 43, 81100 Caserta, Italy

^5^Fondazione Santa Lucia IRCCS, Via del Fosso di Fiorano 64, 00143 Rome, Italy;

^6^Unit of Cell and Developmental Biology, Department of Biology, University of Pisa, Pisa, Italy;

^7^University of Rome 'Tor Vergata', 00133 Rome, Italy

^8^Bioinformatics Unit, Fondazione IRCCS Casa Sollievo della Sofferenza, 71013 San Giovanni Rotondo, Foggia, Italy;

^9^Center for Neuroscience and Cognitive Systems @UniTn, Istituto Italiano di Tecnologia, Rovereto, Italy;

# authors contributed equally

*co-corresponding authors: [usiello@ceinge.unina.it](mailto:usiello@ceinge.unina.it), Department of Environmental, Biological and Pharmaceutical Sciences and Technologies, Second University of Campania, L. Vanvitelli, Via A. Vivaldi, 4, 81100 Caserta, Italy; [napolitano@ceinge.unina.it](mailto:napolitano@ceinge.unina.it), CEINGE Biotecnologie Avanzate, Via G. Salvatore, 48, 80145, Naples, Italy.

Running title: Rhes controls cocaine–related effects.

**Materials and methods**

*Animals:* In this study 10- to 12-week-old wild-type (WT) and Rhes knockout (KO) male mice without a PGK-neo cassette ^1,2^, derived from mating of heterozygous animals and backcrossed to the F11 generation of the C57BL/6J strain, were used. Mice were housed in groups in standard cages (29×17.5×12.5cm) at constant temperature (22±1°C) and maintained on a 12/12-h light/dark cycle, with food and water *ad libitum*.

*Cocaine-induced acute motor response:* Mice were habituated to the experimental cage (35×25×30cm) for 60 min, injected with cocaine (7.5, 15, or 30 mg/kg) or vehicle, and returned to the same cage. The horizontal motor activity was evaluated at 10-min intervals over a 60-min test session through a computerized video tracking system (Videotrack, Viewpoint S.A.).

*Motor responses induced by chronic cocaine treatment:* Male and female mice were injected with cocaine (15 mg/kg) or vehicle for 10 consecutive days. Horizontal motor activity was evaluated at day 1, 5, and 10 and expressed as distance travelled (cm), measured every 10 min over a 60-min test session.

*Cocaine-induced locomotor sensitization:* In a modified version of the cocaine sensitization protocol ^3^, male and female mice were treated with cocaine (15 mg/kg) or vehicle once a day for ten consecutive days and exposed to 21 days of withdrawal. Thereafter, the drug-treated mice were challenged with 7.5 mg/kg cocaine, and the vehicle-treated group was given 7.5 mg/kg cocaine or vehicle. Locomotion was expressed as distance travelled (cm), measured every 10 min over a 60-min test session.

*Cocaine conditioned place preference*

The CPP apparatus consisted of two compartments (20x20x50cm) and a central alley separated by a guillotine door. The two chambers were different for tactile stimuli due to floor textures, one smooth and the other grooved. On day 1 the mice were habituated to the entire apparatus. Each experiment consisted of three phases. Spontaneous preference was evaluated in the pre-conditioning phase, consisting of a session during which each mouse was given free access to both compartments of the apparatus for 15 min. The time spent in each compartment during the pre-conditioning phase was used to determine individual unconditioned preferences. During the conditioning phase, mice were treated with cocaine 2.5 mg/kg, 7.5 mg/kg or vehicle, and placed for 20 min in the ‘non-preferred’ compartment. On the next day, the animals received saline and were placed in the ‘preferred compartment’. This procedure was repeated for 8 days so that each animal received a total of 4 injections with cocaine paired to one specific compartment. Control animals were injected with saline during the entire conditioning phase. During the post-conditioning phase, mice had free access to both compartments and the time spent by each animal in the cocaine-paired compartment was recorded during 15min. The difference in seconds between the time spent in the drug-paired compartment and that spent in the pre-conditioning test was considered as the degree of cocaine-induced conditioning.

*Effect of Rapamycin on locomotor activity*

WT and KO male animals were pre-treated with rapamycin 5mg/kg or vehicle, and placed in the experimental cage (35×25×30cm). One hour after the first administration mice that had been injected with rapamycin or vehicle were treated with cocaine at the dose of 15mg/kg. Finally, the animals injected with vehicle were treated with vehicle or rapamycin 5mg/kg. In the cocaine sensitization protocol, mice were treated with cocaine (15 mg/kg) or vehicle once a day for ten consecutive days, and exposed to 21 days of withdrawal. Thereafter, cocaine-treated mice were challenged with 7.5 mg/kg cocaine 1 hour after 5 mg/kg rapamycin administration. Locomotion was expressed as distance travelled (cm), measured every 10 min over a 60-min test session.

*Combined immunohistochemistry (IHC) and in situ hybridization* *(ISH):* Combined IHC and ISH were performed as described by Pinna and collaborators ^4^ (See supplementary information).

*Combined immunohistochemistry (IHC) and in situ hybridization* *(ISH)*: The animals were perfused transcardially with 4% paraformaldehyde (PFA), and 50-μm-thick brain sections were obtained with a vibratome (Leica Microsystems). Free-floating sections were incubated overnight at 4 °C with a primary mouse anti-TH antibody (1:400, Merk-Millipore, Germany), followed by a 2-hour incubation at room temperature with an Alexa Fluor 488 goat anti-mouse antibody (1:500, Molecular Probes, Eugene, Oregon, USA). Following IHC, ISH was performed using a fluorescein-labeled *Rhes* antisense riboprobe (0.5 Kb, ^5^). A chromogenic reaction using HNPP/Fast Red Fluorescent Detection Set (Roche) was performed to visualize the fluorescein-labeled riboprobe, taking care to avoid quenching the Alexa Fluor 488 fluorescence masking the TH signal in dopaminergic neurons. Images were taken with a Nikon A1 confocal system.

*Quantitative Real-Time (qRT)-PCR*: Total RNA was purified to eliminate potentially contaminating genomic DNA using recombinant DNase (QIAGEN, Hilden, Germany). A quantity of 500 ng of total RNA of each sample was reverse-transcribed with Quanti Tect Reverse Transcription (QIAGEN, Hilden, Germany) using oligo-dT and random primers according to the manufacturer’s instructions. Quantitative PCR (qPCR) amplifications were performed using LightCycler 480 SYBR Green I Master (Roche Diagnostic, Risch, Switzerland) in a LightCycler480 Real Time thermocycler (Roche Diagnostic, Risch, Switzerland). The following protocol was used: 10 s for initial denaturation at 95°C followed by 40 cycles consisting of 10 s at 94°C for denaturation, 10 s at 60°C for annealing, and 6 s for elongation at 72°C. The following primers were used for mouse Rhes cDNA amplification: Rhes forward 5-TCTGGCAACCACCCATTC-3 and Rhes reverse 5-AGGCTGAACACCAGGATGAA-3. β-actin and PP1A were used as housekeeping genes for PCR: β-actin forward 5-CTAAGGCCAACCGTGAAAAG-3 and  β-actin reverse 5-ACCAGAGGCATACAGGGACA-3, PP1A forward 5-GTGGTCTTTGGGAAGGTGAA-3 and PP1A reverse 5-TTACAGGACATTGCGAGCAG-3.

*Western blotting*

Aliquots (2µl) of the homogenate were used for protein determination using a Bio-Rad Protein Assay kit. Equal amounts of total proteins (30 µg) for each sample were loaded on a precast 4-20% gradient gel (BioRad Laboratories, Hercules, California, USA). Proteins were separated by SDS-PAGE and transferred to PVDF membranes (GE Healthcare, Chicago, Illinois, USA) via the Trans Blot Turbo System (BioRad Laboratories, Hercules, California, USA). The blots were incubated with antibodies against phosphorylated forms of GluA1 at the Ser845 residue (pGluA1) (1:500, PhosphoSolution, East Montview Blvd., Aurora, CO, USA). Then, in order to remove primary antibodies, membranes were treated with stripping solution (StripAblot-EuroClone S.p.A-Milan, Italy), according to the manufacturer's protocol. Antibodies against GluA1 (1:500; Merk-Millipore, Germany) were used on the stripped membranes to estimate the total amount of proteins, which were then normalized for GAPDH protein levels using a specific antibody (1:1000; Santa Cruz Biotechnology, Dallas, Texas, USA) for variations in loading and transfer. The expression levels of Rhes protein were obtained by incubating lysates with anti-Rhes antibody (1:1000) ^5^. Rhes levels were normalized by using the antibody against GAPDH (1:1000). All blots were then incubated in horseradish peroxidase-conjugated secondary antibodies and target proteins visualized by ECL detection (Pierce, Rockford, Illinois, USA), followed by quantification through the “Quantity One” software (BioRad Laboratories, Hercules, California, USA) for variations in loading and transfer. Normalized values were then averaged and used for statistical comparisons.

*Midbrain slice preparation*: Male Rhes KO mice and wild-type littermates (2-3 months old) were anesthetized with isoflurane and decapitated. The brain was rapidly removed from the skull and a tissue block containing the midbrain was isolated and immerged in cold artificial cerebrospinal fluid (aCSF) at 8-10 C°. The aCSF contained (in mM): NaCl 126, KCl 2.5, MgCl_2_ 1.2, CaCl_2_ 2.4, NaH_2_PO_4_ 1.2, NaHCO_3_ 24, glucose 10, saturated with 95% O_2_ - 5% CO_2_ (pH 7.4). Horizontal slices (200-250 μm thick) of the ventral midbrain were cut using a vibratome (Leica VT1000S, Leica Microsystems, Wetzlar, Germany). Slices were maintained in aCSF at 33.0 ± 0.5 C° for 30 min before electrophysiological experiments.

*Electrophysiology*: Slices were continuously perfused at 2.0-2.5 ml/min with aCSF. Neurons, visually selected by their morphology, were identified as dopaminergic based on their electrophysiological properties (regular spontaneous firing at 0.5-4 Hz and presence of a large inward current (I_h_) in response to hyperpolarizing voltage steps). Whole-cell patch-clamp recordings were performed with glass borosilicate pipettes (5-8 MΩ) pulled with a PP 83 Narishige puller and filled with a solution containing (in mM): KGluconate 135, KCl 10, CaCl_2_ 0.05, EGTA 0.1, Hepes 10, Na_3_-GTP 0.3, and Mg-ATP 4 (pH 7.35 with KOH). Cells were recorded in voltage-clamp mode (V_H_ = -60 mV). Current signals were filtered at 3 kHz and digitized at 10 kHz using a Multiclamp 700A (Axon Instruments, Foster City, CA) operated by the pClamp10 software (Molecular Devices, Sunnyvale, CA, USA). Amplitudes of membrane currents induced by the D2 agonist quinpirole were calculated by measuring differences between baseline and peak and are expressed as absolute values (pA).

*Constant potential amperometry:* The amperometric signal, which is related to electrically evoked DA release, was recorded in slices of the dorsal striatum ^6^. Briefly, we positioned the DA-recording carbon fiber electrode (diameter 30 μm, length 100 μm, World Precision Instruments) into the striatal slice at a depth of 50–150 μm and near a bipolar Ni/Cr stimulating electrode. The recordings were obtained with the MicroC potentiostat (World Precision Instruments), which maintained the potential between the carbon fiber electrode and the Ag/AgCl pellet at 0.55 V. We electrically stimulated the tissue using a DS3 Stimulator (Digitimer) and a single rectangular pulse was applied every 5 min along a range of stimulation intensities (20–1000 μA, duration 20–40 μs). Signals were digitized with Digidata 1440A coupled to a computer running pClamp 10 (Molecular Devices). Electrode calibration was performed at the end of each experiment by bath-perfused DA (0.3–10 μM). Cocaine ranging from 10 to 100 nM was superfused on striatal slices and its effect was evaluated on the evoked release of DA. Amperometric traces were evaluated in dorsostriatal slices of male WT and KO animals.

*In situ hybridization*

Fresh brain tissue was embedded in Tissue Tek (Sakura), and frozen at -80°C. Using a cryostat, 14-mm-thick sections were cut in the coronal plane and ISH was performed according to protocols using ^35^S-labeled (*Arc*,1.2 Kb; *Zif 268*, 3.0 Kb; *Homer 1a*, 0.6Kb) antisense RNA probes as previously described ^7^. Detailed sequences for each probe are the following: *Arc*, nucleotides 199–1389 (NM_001276684); *Zif 268*, nucleotides 174–3029 (NM_007913.5); and *Homer 1a*, nucleotides 1542–2102 (NM_011982.4). Sections were exposed to Biomax MR X-ray films (Kodak Rochester, New York, USA) for 16 h to four days. Quantification analyses for *in situ hybridization* were performed in a blinded fashion and sample identity was not revealed until correlations were completed. For densitometric analyses, the whole rostrocaudal extent of the prefrontal cortex and the striatum were used and images of autoradiography films resulting from radioactive *in situ hybridization* experiments were scanned at a resolution of 1200 dpi. Optical density (OD) for each gene analyzed was evaluated in the prefrontal cortex and the striatum. Background OD value was determined in structures of the same section devoid of a specific signal and subtracted for correction to obtain the relative OD (ROD) value. Results were expressed as percentage increase/decrease in mRNA expression level for each probe as compared to wild-type, vehicle-treated animals.

*Proteomic studies*: Tissues were lysed in ice-cold lysis buffer (100 mM Triethylammonium bicarbonate TEAB, SDS 1%) and disrupted by two cycles of sonication at a 20% amplitude for 30 s on ice. Lysates were cleared by centrifugation at 16,000 × *g* for 15 min at 4°C. Supernatants were transferred to new tubes, treated with 1 Unit of RQ1 DNase (Promega) for 1 h at room temperature, and the protein concentration determined by using the Pierce BCA Protein assay kit (Thermo Scientific, Rockford, Illinois, USA). For each condition, equal amounts of proteins (50 µg in 100 µL of 100 mM TEAB) were reduced with 10 mM Tris-(2-carboxyethyl)-phosphine (TCEP) for 1 h at 55 °C and alkylated with 18 mM iodoacetamide by incubating samples for 30 min at room temperature in the dark. Proteins were then precipitated overnight by adding six volumes of prechilled acetone. Following centrifugation at 8,000 × *g* for 10 min at 4°C, protein pellets were resuspended in 100 µL of 100 mM TEAB and digested overnight with MS grade trypsin (Pierce, Thermo Scientific, Rockford, Illinois, USA) at an enzyme/substrate ratio of 1:40 at 37°C. Resulting peptide mixtures were chemically labeled with the TMT isobaric tags as previously reported ^8,9^ using the following tags: 126 DStr WT/ NAc WT, 127N DStr KO/ NAc KO, 128C DStr WT 3h/ NAc WT 3h, 129N DStr WT 6h/ NAc WT 6h, 130C DStr KO 3h/ NAc KO 3h, and 131 DStr KO 6h/ NAc KO 6h. Briefly, 0.8 mg of TMT reagents in 41 µL of anhydrous acetonitrile were added to each sample. The reaction proceeded for 1 h and was then quenched for 15 min with hydroxylamine to a final concentration of 0.3%. Two mixtures of samples were then prepared by combining the six conditions for both DStr and NAc tissues at equal amounts. Mixed samples were diluted in 0.1% TFA/2% CH3CN to a final concentration of 0.5 µg/µL for LC-MS analyses.

*High-resolution nanoLC−Tandem Mass Spectrometry:* Aliquots of TMT-labeled samples (2.5 µg) were analyzed by high-resolution nanoLC−tandem mass spectrometry using a Q-Exactive Orbitrap mass spectrometer equipped with an EASY-Spray nanoelectrospray ion source (Thermo Scientific, Rockford, Illinois, USA) and coupled to a Thermo Scientific Dionex UltiMate 3000RSLC nano system (Thermo Scientific, Rockford, Illinois, USA). Solvent composition was 0.1% formic acid in water (solvent A) and 0.1% formic acid in acetonitrile (solvent B). Peptides were loaded on a trapping PepMap™100 μCartridge Column C18 (300 μm x 0.5 cm, 5 μm, 100 Å) and desalted with solvent A for 3 min at a flow rate of 10 μL/min. After trapping, eluted peptides were separated on an EASY-Spray analytical column (50 cm x 75 μm ID PepMap RSLC C18, 3 μm, 100 Angstrom) heated to 35 °C at a flow rate of 300 nL/min, applying the following gradient: 5% B for 3 min, from 5% to 27.5% B in 222 min, from 27.5% to 40% B in 10 min, and from 40% to 95% B in 1 min. Washing (95% B for 4 min) and re-equilibration (5% B for 24 min) steps were always included at the end of the gradient. Eluting peptides were analyzed on the Q-Exactive mass spectrometer operating in positive polarity mode with a capillary temperature of 280 °C and a potential of 1.9 kV applied to the capillary probe ^9^. Full MS survey scan resolution was set to 70000 with an automatic gain control (AGC) target value of 3×10^6^ for a scan range of 375−1500 m/z and maximum ion injection time (IT) of 60 ms. The mass (m/z) 445.12003 was used as lock mass. A data-dependent top 12 method was operated, during which high-energy collisional dissociation (HCD) spectra were obtained at 35000 MS2 resolution with an AGC target of 1 × 10^5^ for a scan range of 200−2000 m/z, maximum IT of 120 ms, 1.6 m/z isolation width, and normalized collisional energy (NCE) of 32. Precursor ions targeted for HCD were dynamically excluded for 30 s. Full scans and Orbitrap MS/MS scans were acquired in profile mode, whereas ion trap mass spectra were acquired in centroid mode. Charge state recognition was enabled by excluding unassigned and 1, 7, 8, >8 charged states. All data were acquired with Xcalibur 3.1 software (Thermo Scientific, Rockford, Illinois, USA).

*Protein Identification and Quantitation:* For data processing, the acquired raw files were analyzed with the Thermo Scientific Proteome Discoverer 2.1 software (Thermo Scientific, Rockford, Illinois, USA) using the SEQUEST HT search engine. The HCD MS/MS spectra were searched against the *Mus musculus* database (version 2015-11-11, number of entries 24771 sequences) assuming trypsin (Full) as digestion enzyme and two missed cleavage sites being allowed. Mass tolerances were set to 10 ppm and 0.02 Da for precursor and fragment ions, respectively. Oxidation of methionine (+15.995 Da) was set as dynamic modification. Carbamidomethylation of cysteine (+57.021 Da) and the TMT label on lysines and the N-terminus (229.1629) were set as static modifications. False-discovery rates (FDRs) for peptide spectral matches (PSMs) were calculated and filtered using the Percolator node in Proteome Discoverer that was run with the following settings: Maximum Delta Cn 0.05, a strict target FDR of 0.01, a relaxed target FDR of 0.05, and validation based on q-value. Protein identifications were accepted when the protein FDR was below 1 % and when present in at least two out of three replicate injections with at least two peptides.

*Bioinformatic analyses:* Enrichment analyses of differentially expressed proteins were performed using Ingenuity Pathway Analysis (IPA) software. For each comparison, the top 20 enriched *diseases and functions* categories were extracted, according to the 20 highest –log10(p-value) values. The enriched Gene Ontology (GO) molecular functions terms were extracted by using ToppFun tool of the ToppGene Suite ([http://toppgene.cchmc.org](http://toppgene.cchmc.org/)). Benjamini-Hochberg adjusted p-values were considered significant if <0.05. Heatmaps and enrichment results were drawn through pheatmap (<https://CRAN.R-project.org/package=pheatmap>) and ggplot2 packages ^10^, respectively, of the R 3.4.2 software ([http://www.R-project.org](http://www.R-project.org/)). Finally, protein interactions were checked against the Ingenuity Knowledge Base and served as *seeds* for generating networks. Networks were scored based on the number of network-eligible molecules they contained. Scores were based on the hypergeometric distribution and were calculated with the right-tailed Fisher’s exact test. The higher the score, the lower was the probability of finding the observed number of network-eligible molecules in a given network by chance.

**FIGURES**

**
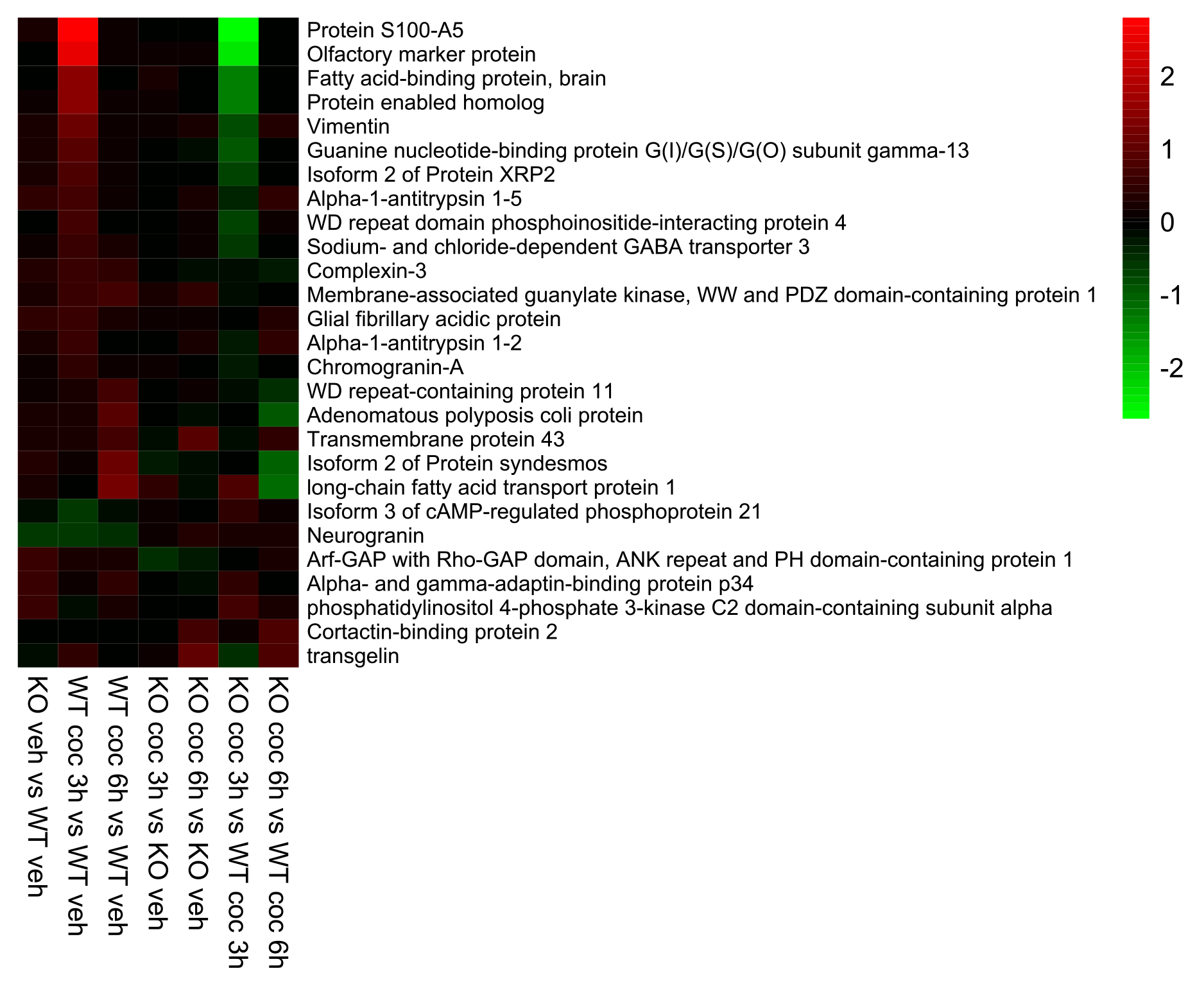
**

**Figure S1. Heatmap of differentially expressed proteins in the NAc tissue samples.**

Down-regulated and up-regulated proteins are coloured in green and red respectively. Colour gradients represent the strength of differential expression.


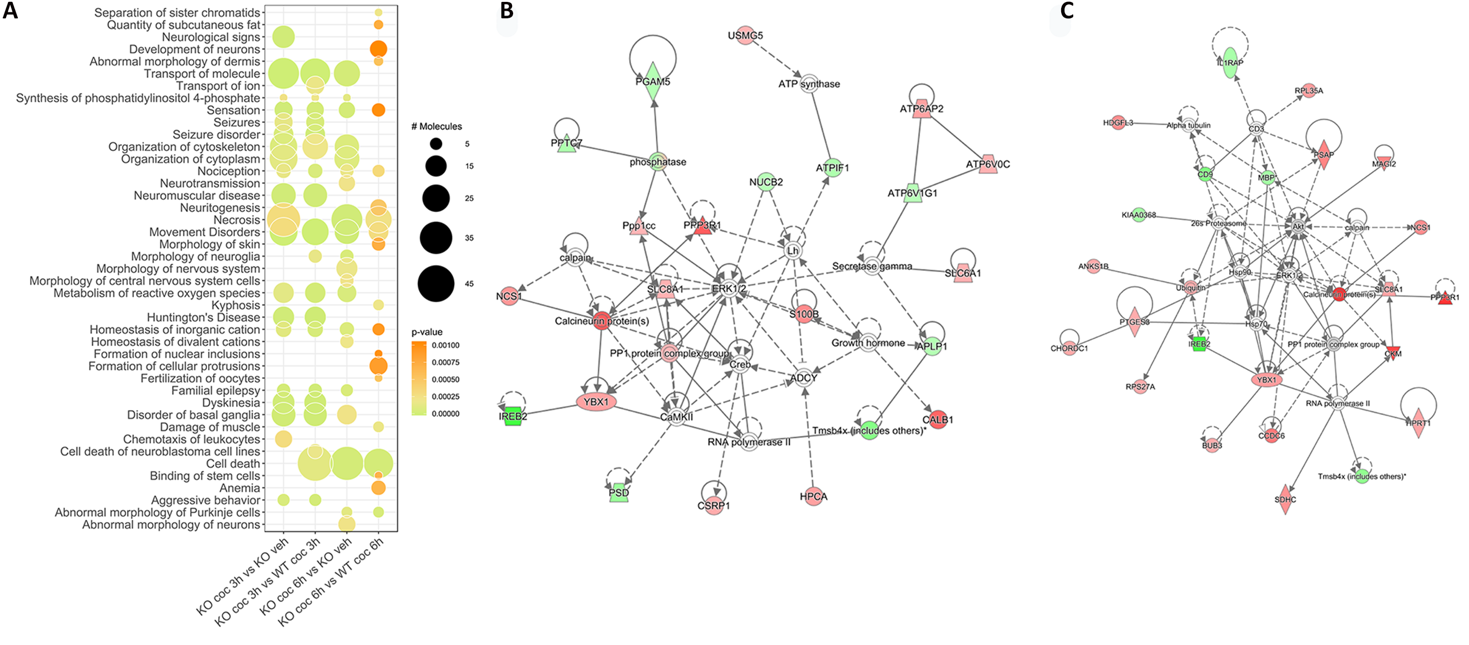


**Figure S2.** Bubble plot of enriched “Disease and Functions” categories in dorsal striatum upon cocaine administration, and nteraction network of differentially expressed proteins in dorsal striatum upon cocaine administration. **A** Bubble plot of top 20 enriched “Disease and Functions” categories obtained through IPA software for comparisons relative to KO DStr samples treated with cocaine at 3h and 6h with respect to both untreated Rhes KO and wild type samples at the same time points of treatment. Bubble sizes are proportional to the number of differentially expressed proteins belonging to the enriched functions. Colour gradients represent p-values. **B,C** To determine the downstream pathway(s) triggered by cocaine treatment upon Rhes ablation, network maps including differentially regulated proteins identified by MS analysis were constructed in silico by using the IPA software. Protein interaction network included a subset of DStr differentially expressed proteins in KO mice, treated with cocaine at 3h (B) and 6h (C) with respect to wild type samples. According to IPA categorization, proteins up-regulated and down-regulated are colored in red and green, respectively. The pathway components identified by the algorithm are reported in white. Molecules are named according to IPA software.


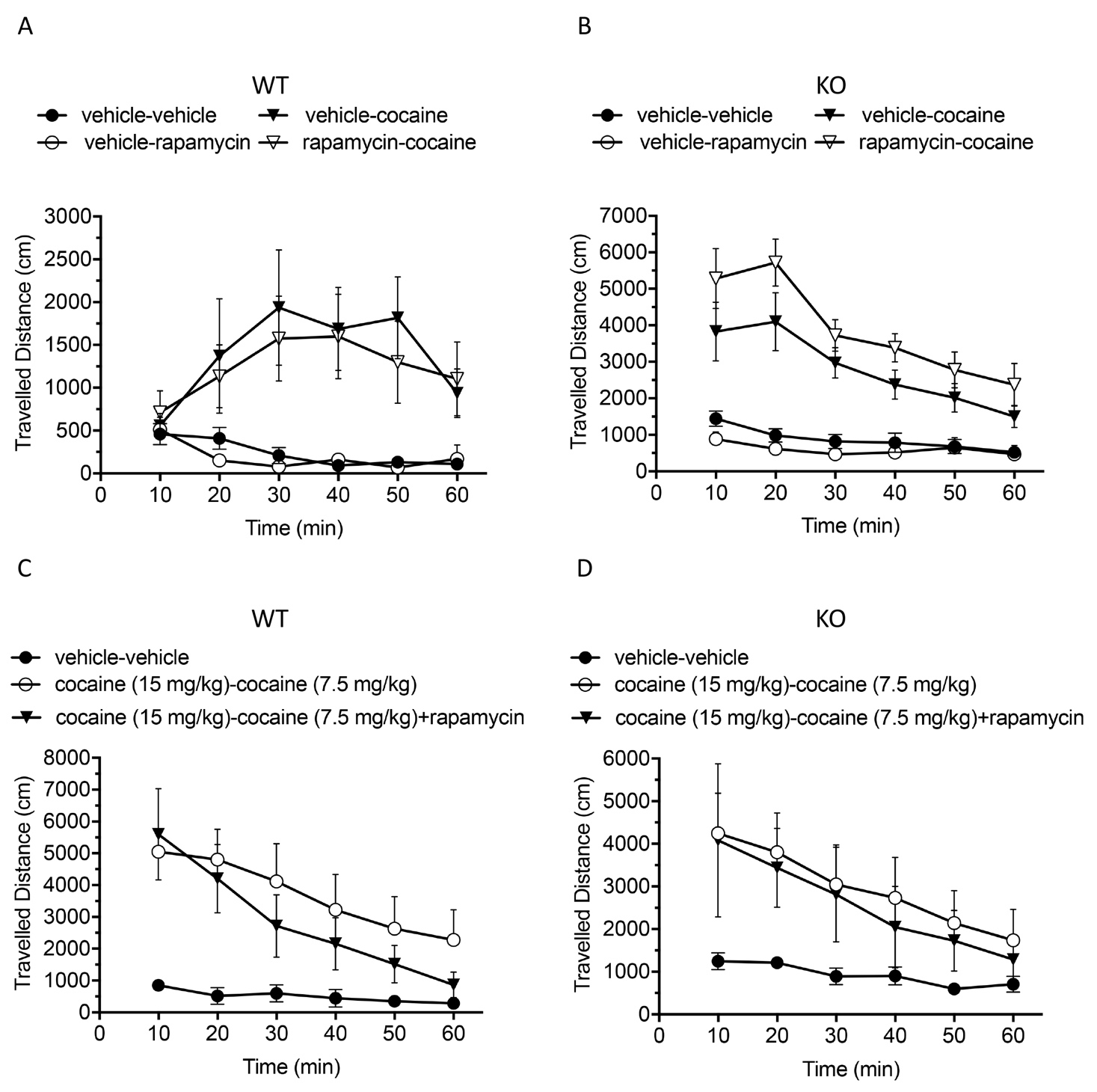


**Figure S3. Effect of rapamycin on the exacerbated motor response induced by the acute cocaine treatment.** (**A,B**) Horizontal motor activity in WT (A) and KO (B) mice, pre-treated with rapamycin at the dose of 5mg/kg or vehicle. One hour after the first administration, mice previously injected with rapamycin were treated with 15 mg/kg cocaine (rapamycin-cocaine group: n = 10/genotype), while animals pre-treated with vehicle were administered cocaine (vehicle-cocaine group: n = 10/genotype), rapamycin 5mg/kg (vehicle-rapamycin group: n = 6/genotype) or vehicle (vehicle-vehicle group: n = 10/genotype). Two-way RM ANOVA within genotypes, treatment effect; WT: F_(3,32)_ = 4.18, *p* = 0.0132; KO: F_(3,32)_ = 20.44, *p* < 0.0001; Uncorrected Fisher’s LSD test; WT: vehicle-vehicle vs vehicle-rapamycin: *p* = 0.9262; vehicle-vehicle vs vehicle-cocaine: *p* = 0.0090; vehicle-cocaine vs rapamycin-cocaine: *p* = 0.7231. Locomotion is expressed as distance travelled (cm), measured every 10 min over a 60-min test session. All values are expressed as mean ± SEM. Genotypes and treatments are as indicated.

**REFERENCEES**

1 Ghiglieri, V. *et al.* Rhes influences striatal cAMP/PKA-dependent signaling and synaptic plasticity in a gender-sensitive fashion. *Sci Rep* **5**, 10933, doi:10.1038/srep10933 (2015).

2 Sciamanna, G. *et al.* Rhes regulates dopamine D2 receptor transmission in striatal cholinergic interneurons. *Neurobiol Dis* **78**, 146-161, doi:10.1016/j.nbd.2015.03.021 (2015).

3 Smith, L. N. *et al.* Fragile X mental retardation protein regulates synaptic and behavioral plasticity to repeated cocaine administration. *Neuron* **82**, 645-658, doi:10.1016/j.neuron.2014.03.028 (2014).

4 Pinna, A. *et al.* The Small GTP-Binding Protein Rhes Influences Nigrostriatal-Dependent Motor Behavior During Aging. *Mov Disord* **31**, 583-589, doi:10.1002/mds.26489 (2016).

5 Spano, D. *et al.* Rhes is involved in striatal function. *Mol Cell Biol* **24**, 5788-5796, doi:10.1128/MCB.24.13.5788-5796.2004 (2004).

6 Napolitano, F. *et al.* Role of aberrant striatal dopamine D1 receptor/cAMP/protein kinase A/DARPP32 signaling in the paradoxical calming effect of amphetamine. *J Neurosci* **30**, 11043-11056, doi:10.1523/JNEUROSCI.1682-10.2010 (2010).

7 Migliarini, S., Pacini, G., Pelosi, B., Lunardi, G. & Pasqualetti, M. Lack of brain serotonin affects postnatal development and serotonergic neuronal circuitry formation. *Mol Psychiatry* **18**, 1106-1118, doi:10.1038/mp.2012.128 (2013).

8 Cimini, D. *et al.* Physiological characterization and quantitative proteomic analyses of metabolically engineered E. coli K4 strains with improved pathways for capsular polysaccharide biosynthesis. *Biotechnol Bioeng* **115**, 1801-1814, doi:10.1002/bit.26597 (2018).

9 Carafa, V. *et al.* RIP1-HAT1-SIRT Complex Identification and Targeting in Treatment and Prevention of Cancer. *Clin Cancer Res* **24**, 2886-2900, doi:10.1158/1078-0432.CCR-17-3081 (2018).

10 Wickham, H. A Layered Grammar of Graphics. *J Comput Graph Stat* **19**, 3-28, doi:10.1198/jcgs.2009.07098 (2010).


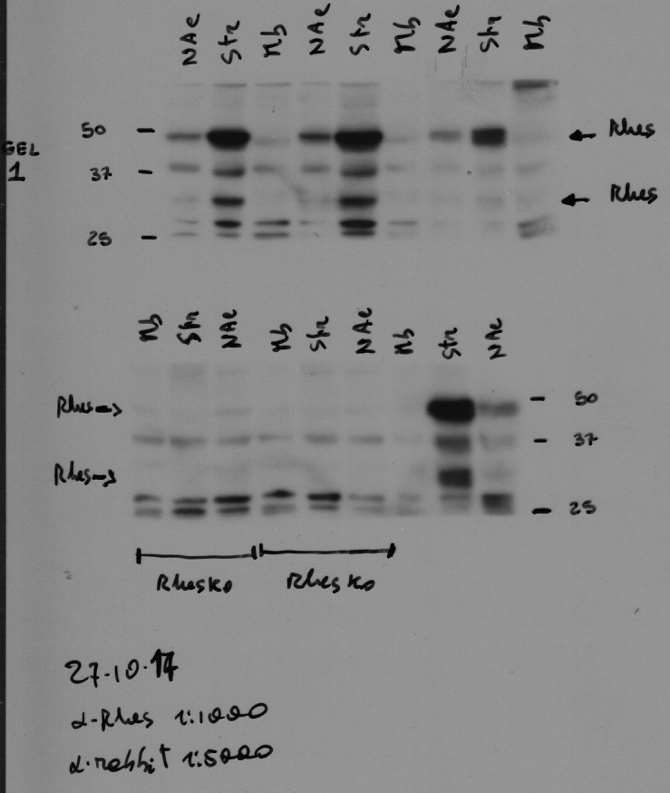

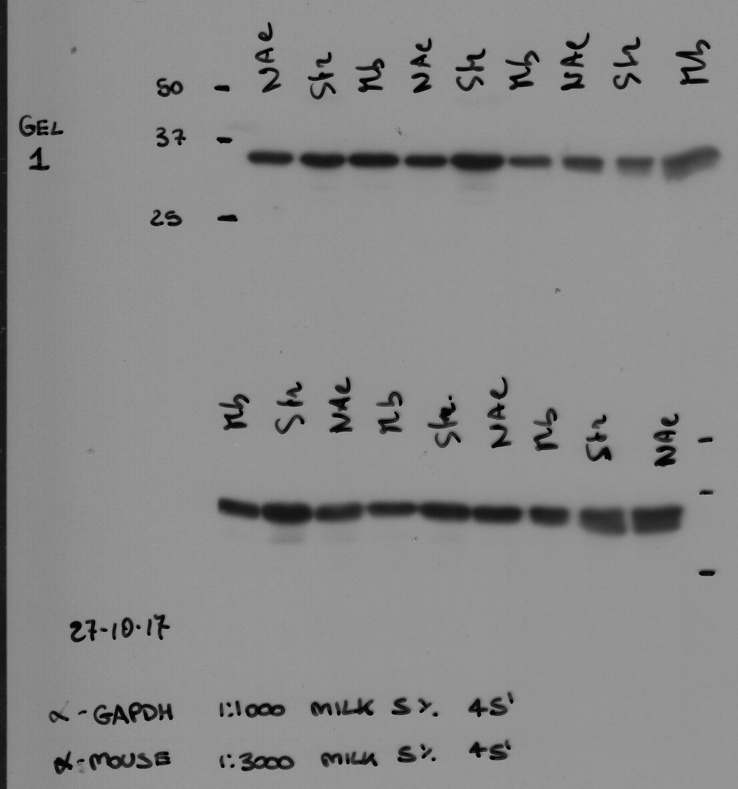


**Full-length gels relative to the analysis of 32 kDa and 47 kDa Rhes protein abundance (left panel), as well as GAPDH levels (right panel), in DStr and NAc from wild-type mouse lysates (Fig. 3B,C).**

**
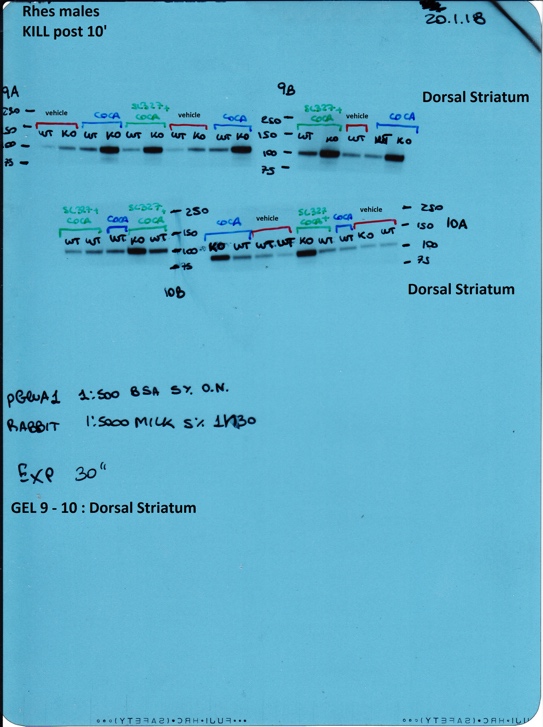

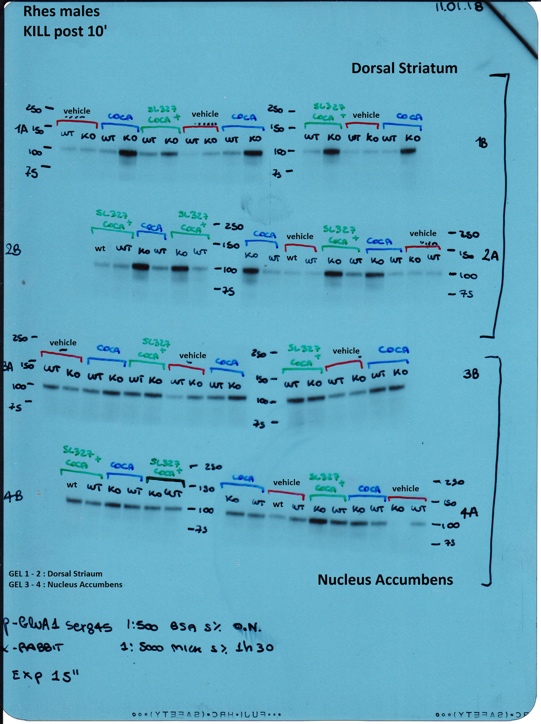
**

**Full-length gels relative to the analysis of pGluA1 levels in DStr and NAc from wild-type mouse lysates, 10min post-cocaine injection (Fig. 3D,D’).**

**
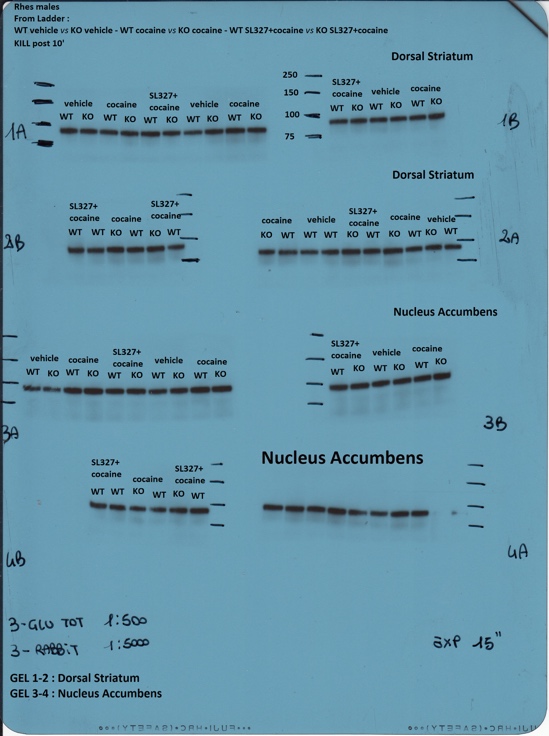

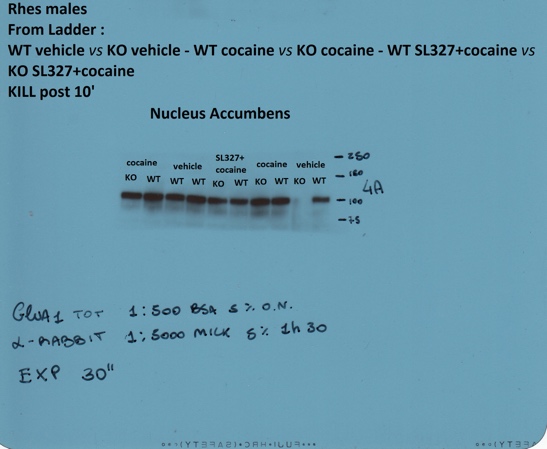
**

**
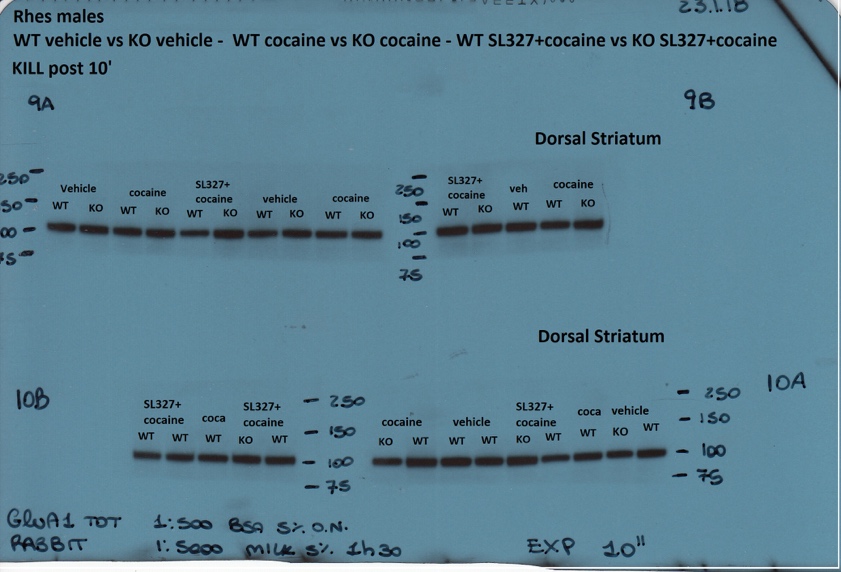
**

**Full-length gels relative to the analysis of GluA1 levels in DStr and NAc from wild-type mouse lysates, 10min post-cocaine injection (Fig. 3D,D’).**

**
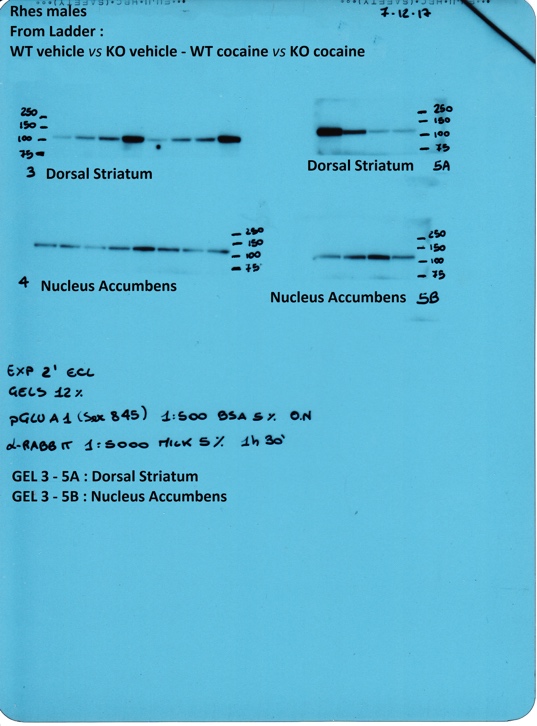

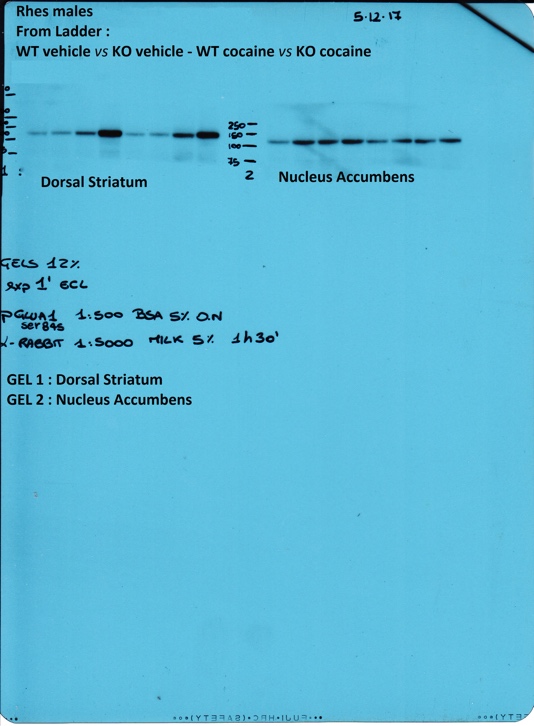

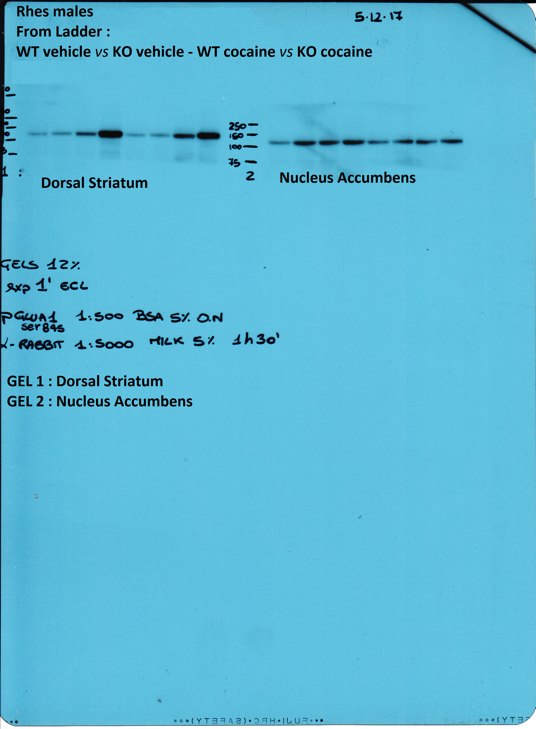
**

**Full-length gels relative to the analysis of GluA1 levels in DStr and NAc from wild-type mouse lysates, 30min post-cocaine injection (Fig. 3E,E’).**

**
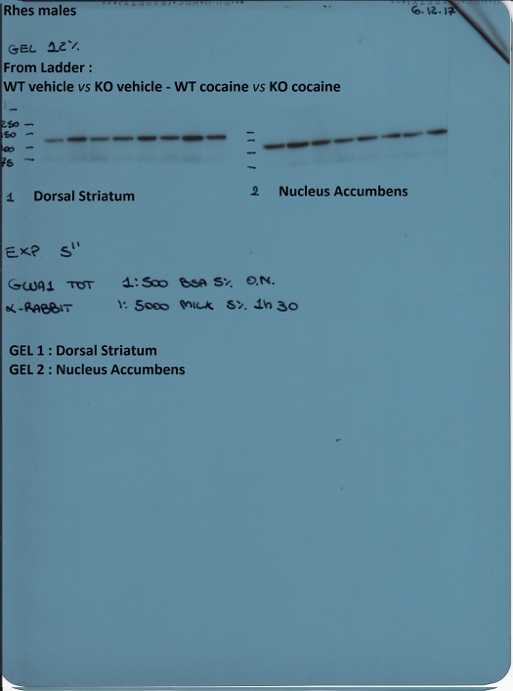

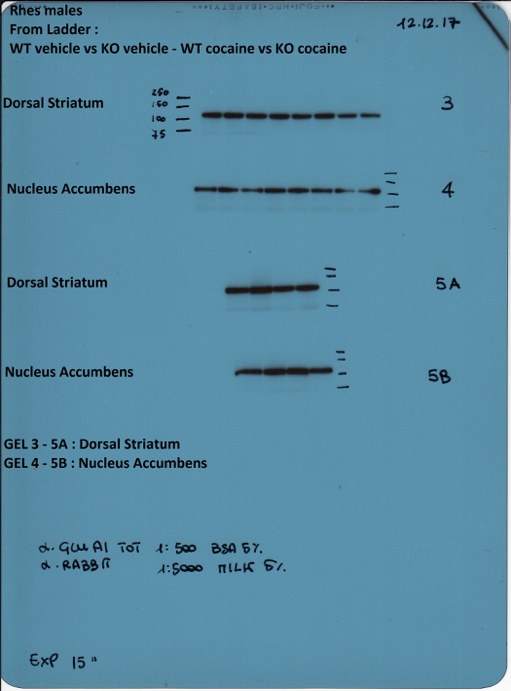
**

**Full-length gels relative to the analysis of GluA1 levels in DStr and NAc from wild-type mouse lysates, 30min post-cocaine injection (Fig. 3E,E’).**

**
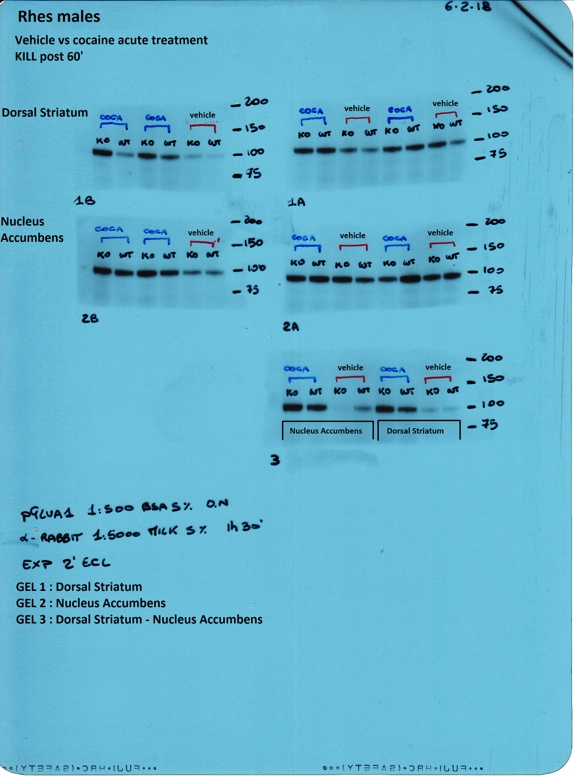

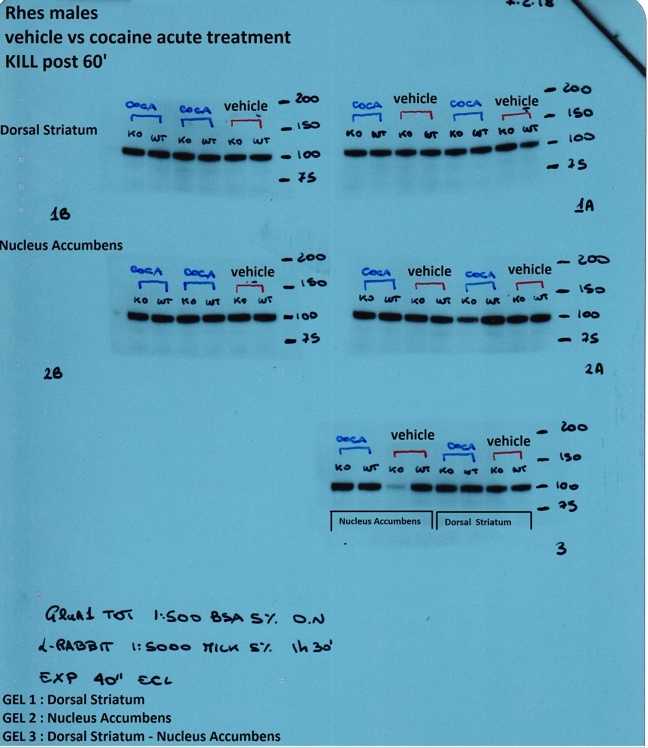
**

**Full-length gels relative to the analysis of pGluA1(left panel) and GluA1 (right panel) levels in DStr and NAc from wild-type mouse lysates, 60min post-cocaine injection (Fig. 3F,F’).**
